# Hypolipidemic and Antihyperlipidemic Effects of *Holarrhena pubescens* Methanolic Extract Is Mediated through Inhibition of Lipase Activity and Lipid Accumulation

**DOI:** 10.3390/life13071435

**Published:** 2023-06-24

**Authors:** AbdulRahman A. I. Alyahya, Mohammed Asad, Mohammed Alrouji, Kamal Eldin Ahmed Abdelsalam, Adel Mashan Rashed Al-Mutairi, Monjid Ahmed Ibrahim Ahmed

**Affiliations:** Department of Clinical Laboratory Science, College of Applied Medical Sciences, Shaqra University, Shaqra 11961, Saudi Arabia; malrouji@su.edu.sa (M.A.); kabdelsalam@su.edu.sa (K.E.A.A.); admaalmutairi@moh.gov.sa (A.M.R.A.-M.); monjid@su.edu.sa (M.A.I.A.)

**Keywords:** cholesterol, triglyceride, lipase, lipid accumulation, DPP-IV

## Abstract

*Holarrhena pubescens* seeds are used in the treatment of various diseases, especially diabetes and associated complications, in different parts of the world. The present study was undertaken to determine the hypolipidemic and antihyperlipidemic effects of methanolic extract of *H. pubescens* seeds in rats. The extract was subjected to LC-MS analysis to determine the chemical constituents. The hypolipidemic action was studied by determining the effect of 28-day oral administration of seed extract on serum cholesterol, serum triglycerides, and serum HDL-cholesterol levels. The antihyperlipidemic action was studied in rats fed with a high-fat diet containing cholesterol and saturated fat, and the same lipid parameters were estimated during 28-day treatment. To elucidate its probable mechanism of action, in vitro studies on the inhibition of lipid accumulation in preadipocytes, DPP-IV inhibitory effect, and lipase enzyme inhibition were studied. The seed extract reduced serum levels of cholesterol and triglycerides in both normal rats and animals fed with a high-fat diet without a significant effect on HDL-cholesterol levels. The seed extract was highly effective in inhibiting lipase enzyme activity but showed a modest effect on the inhibition of lipid accumulation and DPP-IV. The results demonstrated that *H. pubescens* seed extract has hypolipidemic and antihyperlipidemic effects mediated probably through inhibition of lipase enzyme activity.

## 1. Introduction

Herbal drugs are increasingly being investigated for their hypolipidemic actions. A range of hypolipidemic effects have been reported for different herbs with some showing promising effects while a few others have been reported to have adverse effects along with hypolipidemic actions [1]. Some of these herbal formulation such as guggul have made it into the market for commercial use [2]. Other prominent hypolipidemic herbs include fenugreek, nigella, and ginger, to name a few [3]. Despite this, several herbal drugs are being traditionally used without being scientifically proven to have therapeutic effects [4].

*Holarrhena pubescens* is a medicinal plant native to Africa, as well as to the tropical and subtropical areas of Asia. It is a flowering plant native to India, south-central China, Pakistan, Cambodia, Myanmar, Thailand, Vietnam, Nepal, Bhutan, Bangladesh, Laos, Malawi, Mozambique, Kenya, northern Tanzania, Zaire, Zambia, and Zimbabwe [5]. *H. pubescens* Wall. ex G. Don belongs to the kingdom Plantae, order Gentianales, family Apocynaceae, and the genus Holarrhena R. Br. It is often known as Tellicherry bark (English), and it is known commercially in the local market of the Kingdom of Saudi Arabia as the ‘tongue of the bird’. The plant exhibits antimalarial [6], antidiarrheal (Panda et al., 2012), antibacterial [7], and antiamoebic properties [8]. The plant had adverse effects in pregnant or lactating women [9]. We have earlier reported the antidiabetic effect of *H. pubescens* and determined its mechanism of action [10]. It has been claimed by some authors that *H. pubescens* possesses a hypolipidemic effect along with antidiabetic actions [11,12]. However, none of these studies investigated the antihyperlipidemic effect, and no attempt has been made to evaluate the mechanism(s) involved in the hypolipidemic effect. The present study was undertaken to evaluate the hypolipidemic activity of methanolic extract in normal and hyperlipidemic rats, and an attempt was also made to determine the probable mechanism(s) involved in the observed effect. The extract was subjected to LC-MS analysis to identify different chemical constituents present in it.

## 2. Materials and Methods

### 2.1. Materials

Reagents, cholesterol, standard drugs, and biochemical kits for lipid profiles were obtained from different suppliers and were of analytical grade. Saturated fat (brand: Dalda vegetable ghee) was purchased from reputed suppliers in the local market.

### 2.2. Preparation and Liquid Chromatography–Mass Spectrometry Analysis of the Extract

*H. pubescens* was purchased from a local market in Riyadh and validated by Prof. Dr. Hosam Osama Elansary, who specializes in floriculture, ornamental horticulture, and garden design (taxonomist) at the Department of Plant Production, King Saud University. The maceration process was used to make the extract [13]. The obtained extract was evaporated in a vacuum to obtain residues of the active ingredient of the extract.

A LC-MS analysis was carried using a liquid chromatography system (SCL-40, Shimadzu, Japan) composed of a pump (LC-40B X3) with an LC stop time of 15 min. A C18 column (150 × 2.1 mm) was used, and the column temperature was set at 40 °C. A binary gradient mode was used for the separation of the constituents with a flow rate of 0.3 mL/min. Acetonitrile and ammonium formate buffer were used as solvents. The maximum pressure was set at 1020 kgf/cm^2^. Mass spectra were obtained using a triple quadropole mass spectrometer (LCMC-8050). Mass spectra were recorded in negative and positive ionization modes between 100 and 1000 *m*/*z*.

### 2.3. Animals

Adult male Sprague–Dawley rats weighing between 176 g and 196 g were used. The rats were acclimatized to the defined environmental characteristics, which included temperature (24 ± 1 °C), relative humidity (60 ± 10%), and a 12-hour day–night cycle. The experimental environment of the animal housing was maintained until the experiment was completed. The animals were fed rat chow that contained all important nutrients and trace elements (Table 1) or with a high-fat diet as mentioned in the experimental protocol below. The ethical research committee of Shaqra University authorized the study protocol under agreement number (Registration No. ERC SU 20220018).

### 2.4. Hypolipidemic Activity

The animals were separated into four groups consisting of six animals each: the first group served as control, the second group was treated with a known hypolipidemic drug- losartan (10 mg/kg, p.o) [14], and the third and fourth groups received *H. pubescens* extract orally at two different doses of 250 mg/kg and 500 mg/kg [10]. The treatment was given for 28 days, and the serum levels of cholesterol, HDL-cholesterol, and triglycerides were determined using an automated analyzer (Roche, St. Louis, MO, USA).

### 2.5. Anti-Hyperlipidemic Effect

In this experiment, there were five groups of animals with two control groups; group I consisted of normal animals, while group II was animals fed with a high-fat diet containing standard rat chow—67%, vegetable saturated fat (Dalda brand)—30%, and cholesterol powder—3% [15]. The third group of animals was on a high-fat diet and received losartan (10 mg/kg, p.o). Similarly, the last two groups were on a high-fat diet and received *H. pubescens* extract orally at two different doses of 250 mg/kg and 500 mg/kg. The animals of groups III to V were on a high-fat diet for two weeks. The drug treatment started from the beginning of the third week and continued for 28 days while maintaining the high-fat diet. The serum levels of cholesterol, HDL-cholesterol, and triglycerides were determined using an autoanalyzer that gave results in mmol/L, and this was converted into mg/dl by multiplying with 38.67 [16].

### 2.6. Lipid Accumulation in 3T3L1-Preadipocytes

We seeded 1000 μL of 3T3L1 preadipocytes cell suspension in a 24-well plate (6000 cells per well) with 5% CO_2_ at 37 °C to grow for three days. Following this, 1000 μL of the *H. pubescens* extract at concentrations of 31.25, 62.5, and 125 μg/mL and positive control (Rosiglitazone; 0.4 μg/mL) were added. The oil-red staining method was used for the lipid accumulation assay [17].

### 2.7. Dipeptidyl Peptidase-4 (DPP-IV) Inhibition

*H. pubescens* extract (31.25, 62.5, and 125 μg/mL) or diprotin A (50 μg/mL) was mixed with human recombinant DPP-IV enzyme solution in Tris buffer. The ρNA substrate (Gly-Pro-ρNA) dissolved in Tris buffer was used to initiate the reaction, and acetic acid was added to stop the reaction. The absorbance was measured at 410 nm [18]. The percentage inhibition was calculated using the following equation:

% inhibition = (Mean OD of untreated *−* Mean OD of test/Mean OD of untreated) *×* 100 (1)

### 2.8. Lipase Inhibition Activity

The plant extract or standard (orlistat; 50 μg/mL) or negative control (distilled water) was mixed with porcine pancreatic solution Tris-HCl buffer (pH 8.0) and centrifuged. Substrate solution (pNPP in isopropanol added to Tris-HCl buffer containing gum Arabic, sodium deoxycholate, and Triton X-100) was then added [19]. The absorbance was read at 405 nm. The percent inhibition of enzyme activity by the test sample was calculated by the following Formula (1).

### 2.9. Statistical Analysis

In this statistical analysis, SPSS (version 26) was used to calculate statistical means, SEM, and ANOVA tests (one-way and two-way) were used to measure the variation. *p* value < 0.05 was considered significant.

## 3. Results

### 3.1. Preliminary Phytochemical Analysis

The maceration process for extraction of *H. pubescens* seeds yielded 10.76% *w*/*w* of the extract.

**Figure 1:**
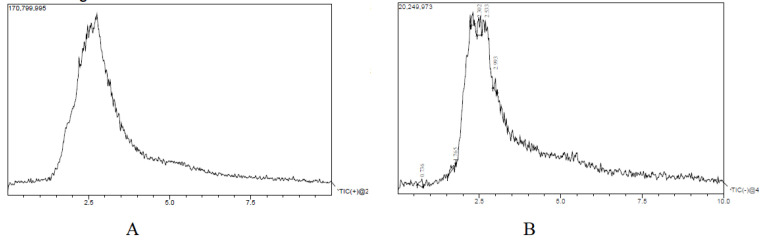
LC-MS analysis of *H. pubescens* extract. Chromatogram in positive mode (**A**); chromatogram in negative mode (**B**) showing different peak. A total of 12 suspected molecules were identified in the positive mode, while in the negative mode, 14 suspected molecules were identified.

The liquid chromatography and mass spectrometry for negative and positive modes data are shown in Figure 1. A number of chemical constituents were detected in both positive and negative modes. The list of chemical constituents identified is given in Table 2. About 14 compounds were identified in the positive mode and 12 chemicals in the negative mode. The mass spectra of the suspected molecules are provided as a Supplementary File (Figure S1).

### 3.2. Hypolipidemic Activity in Normal Animals

**Figure 2:**
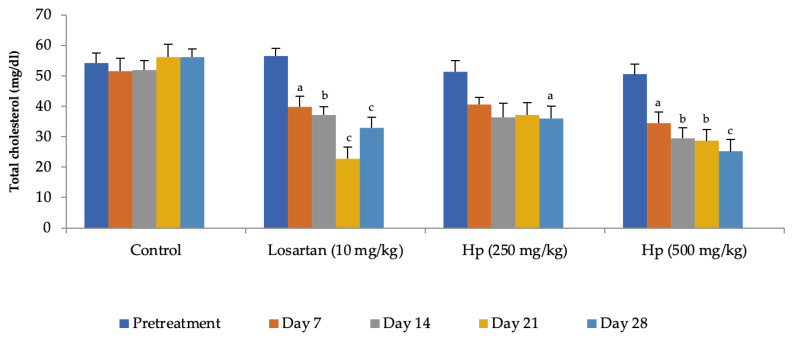
Effect on serum cholesterol levels. All values are mean ± SEM, n = 6, ^a^ *p* < 0.05, ^b^ *p* < 0.01, ^c^ *p* < 0.001 compared to pretreatment values. Hp (*H. pubescens)*. Cholesterol-lowering effect of losartan (10 mg/kg) was observed from Day 7 onward, and maximum effect was observed on Day 21. *H. pubescens* (250 mg/kg) was least effective among the treatments with cholesterol-lowering action seen after 28 days of treatment, while the *H. pubescens* (500 mg/kg) was as effective as losartan (10 mg/kg).

**Figure 3:**
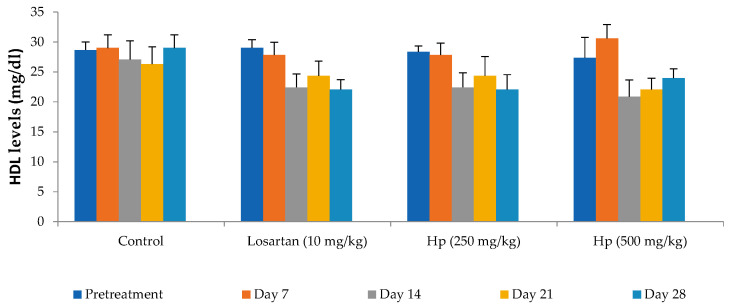
Effect on serum HDL-cholesterol levels. All values are mean ± SEM, n = 6, values were not statistically different when compared with pretreatment values. Hp (*H. pubescens)*. Losartan (10 mg/kg), *H. pubescens* at both doses did not affect serum HDL levels significantly at the tested doses. Surprisingly, a nonsignificant reduction after treatment with losartan (10 mg/kg) and *H. pubescens* (250 mg/kg), while with *H. pubescens* (500 mg/kg), the effect was erratic.

**Figure 4:**
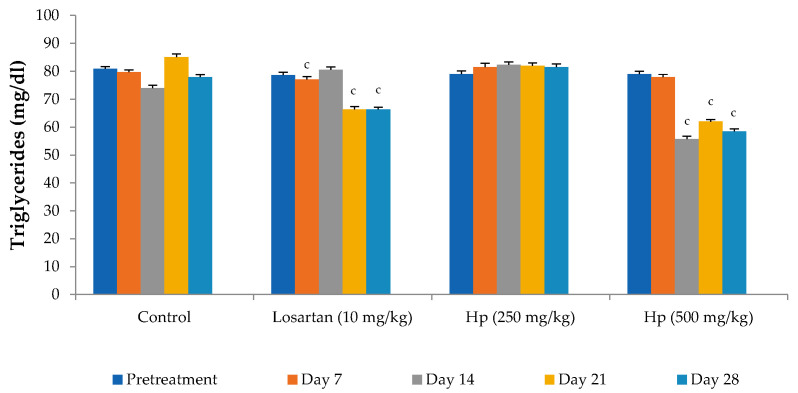
Effect on serum triglyceride levels. All values are mean ± SEM, n = 6, ^c^ *p* < 0.001 compared to pretreatment values. Hp (*H. pubescens)*. The effect on serum triglyceride levels was more evident in the *H. pubescens* (500 mg/kg) group as compared to losartan. *H. pubescens* (250 mg/kg) did not produce any significant change in serum triglyceride levels.

Administration of losartan or *H. pubescens* extract for four weeks showed varying effects in different groups. Losartan (10 mg/kg, p.o) reduced the serum cholesterol levels from the 7th day of treatment, and its effect gradually increased with the treatment period till the 28th day when compared to pretreatment values (*p* < 0.001). The lower dose of *H. pubescens* extract (250 mg/kg, p.o) showed its cholesterol-lowering effect after 28 days of treatment, while the higher dose of the extract revealed its cholesterol-lowering potential from Day 7 onward. No significant difference was observed between lower and higher dose extract treatment (Figure 2). Despite a reduction in the serum levels of total cholesterol, no significant change in the serum levels of HDL-cholesterol was observed in any of the treatment groups when compared to pretreatment values (Figure 3). Determination of the effect on serum triglycerides revealed that the lower dose of *H. pubescens* extract (250 mg/kg, p.o) was ineffective in changing serum triglycerides levels in normal animals, while the higher dose of *H. pubescens* extract (500 mg/kg, p.o) was highly effective in bringing down the serum triglycerides when compared to pretreatment values (*p* < 0.001). Losartan produced a significant reduction in serum triglycerides from Day 14 onward (Figure 4).

### 3.3. Antihyperlipidemic Effect

**Figure 5:**
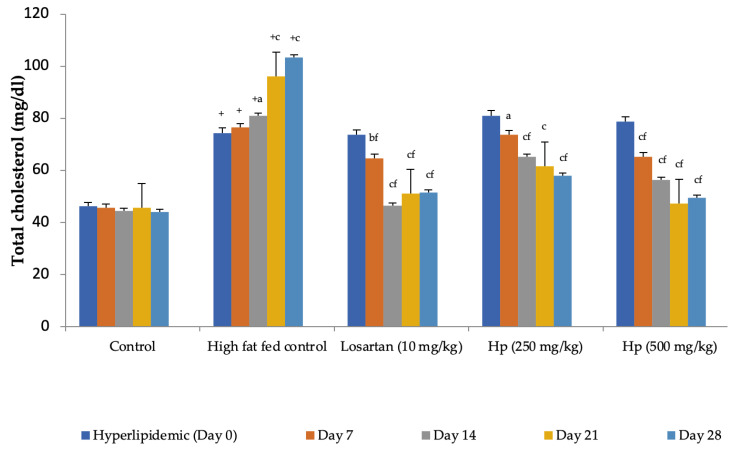
Effect on serum cholesterol levels in hyperlipidemic rats. All values are mean ± SEM, n = 6, ^+^ *p* < 0.001 compared to control, ^a^ *p* < 0.05, ^b^ *p* < 0.01, ^c^ *p* < 0.001 compared to pretreatment values, ^f^ *p* < 0.001 compared to high-fat fed control at the same week. Hp (*H. pubescens)*. Continuous feeding of high-fat diet raised the serum cholesterol levels significantly in high-fat diet control compared to control. *H. pubescens* (500 mg/kg) showed a comparable hypocholesterolemic effect with losartan (10 mg/kg).

**Figure 6:**
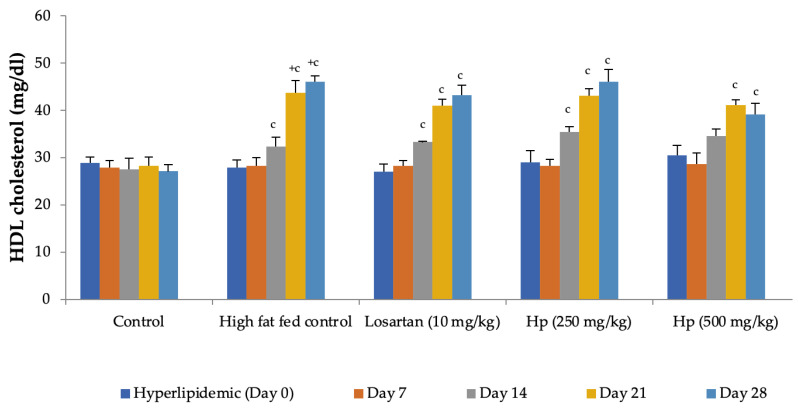
Effect on serum HDL-cholesterol levels in hyperlipidemic rats. All values are mean ± SEM, n = 6, ^+^ *p* < 0.001 compared to control, ^c^ *p* < 0.001 compared to pretreatment values. Hp (*H. pubescens)*. The effect on serum HDL levels was not significant when treatments were compared with high-fat fed control animals.

**Figure 7:**
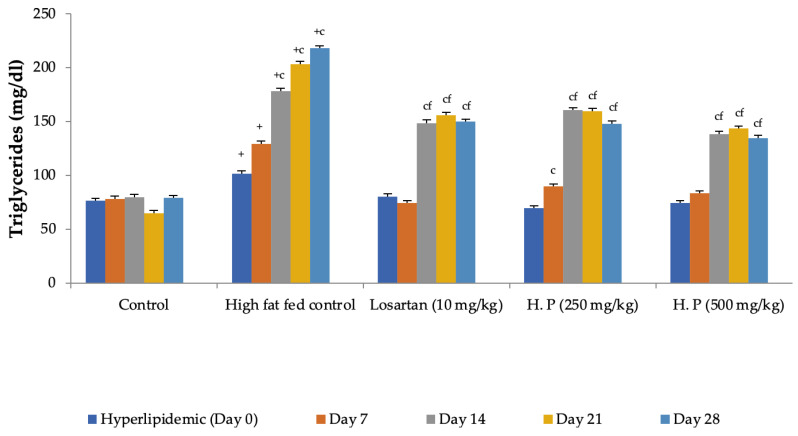
Effect on serum triglycerides levels in hyperlipidemic rats. All values are mean ± SEM, n = 6, ^+^ *p* < 0.001 compared to control, ^c^ *p* < 0.001 compared to pretreatment values, ^f^ *p* < 0.001 compared to high-fat fed control at the same week. Hp (*H. pubescens)*. All treatment showed significant reduction in the serum triglyceride levels when compared to high-fat fed control.

Feeding animals with a high-fat diet for two weeks before the treatment began increased the pretreatment values of cholesterol in group II to group IV animals as compared to control (group I). Feeding of a high-fat diet during the treatment to rats in the high-fat diet control group continued to increase serum cholesterol levels with a significant increase observed from Day 14 onward as compared to Day 0. In contrast, a significant reduction in serum cholesterol levels was observed in all treatment groups receiving the high-fat diet along with losartan or *H. pubescens* extract, indicating their excellent abilities to reduce serum cholesterol (Figure 5). Determination of the effect on serum HDL-cholesterol levels revealed an increase in their levels in high-fat diet control, losartan, and the lower dose of *H. pubescens* extract (250 mg/kg), while no such effect was observed in animals that received the higher dose of *H. pubescens* extract (500 mg/kg), suggesting an unusual non-dose-dependent effect (Figure 6). Unlike the effect on serum cholesterol levels, where all the treatments reduced the serum cholesterol levels despite animals being fed a high-fat diet, the levels of serum triglycerides continued to rise in all the treatment groups, though the percentage increase in losartan and extract treatment groups were less as compared to the high-fat diet control. The serum triglycerides rose continuously in the high-fat diet control and reached about 300% of its value on Day 0, while in the losartan group, the serum triglycerides on Day 28 were at 186% of its value at Day 0. The triglyceride levels on Day 28 in lower- and higher-dose extract treatments were at 213% and 183%, respectively. A comparison of the effect of treatment groups with the high-fat fed group showed that a significant reduction in serum triglycerides was observed from Day 14 onward in all the treatment groups in the same week (Figure 7).

### 3.4. Lipid Accumulation in 3T3L1-Preadipocytes Cell Line

**Figure 8:**
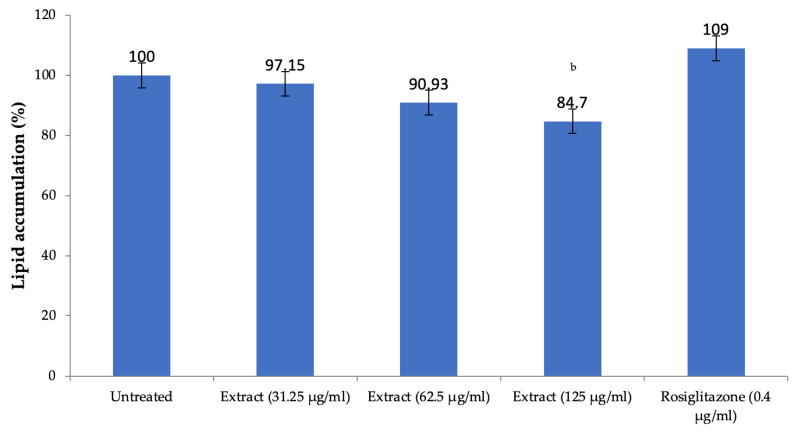
Lipid accumulation in 3T3L1 preadipocytes in-vitro. All values are mean ± SEM, n = 3, ^b^ *p* < 0.01 compared to untreated cells. A dose-dependent decrease in the lipid accumulation in the preadipocytes was observed after treatment with *H. pubescens* extract. However, the effect was less, and it was significant only at the highest tested concentration of 125 µg/mL.

The extract of *H. pubescens* decreased the lipid accumulation in 3T3L1 preadipocytes in a dose-dependent manner with significant reduction observed at a concentration of 125 µg/mL as compared to untreated cells (*p* < 0.01). As expected, rosiglitazone used as positive control increased lipid accumulation, but the effect was not significant (Figure 8).

### 3.5. DPP-IV Inhibition Activity

**Figure 9:**
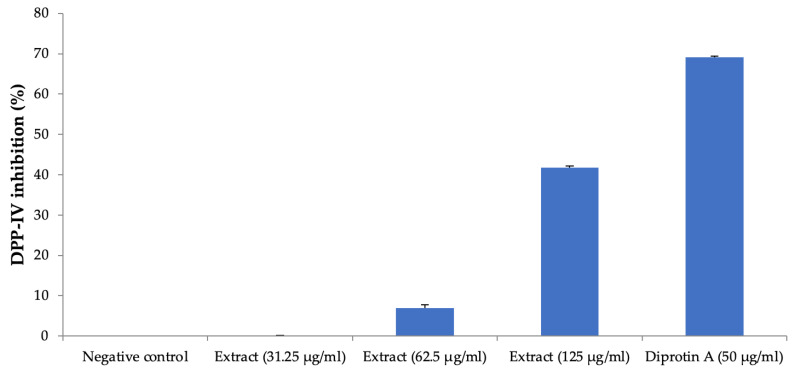
DPP-IV inhibition activity. All values are mean ± SEM, n = 3. The effect on DPP-IV inhibition was weak, and *H. pubescens* extract showed about 42% inhibition of DPP-IV at the highest concentration of 125 µg/mL. Diprotin A showed a good inhibition of 69% at the tested concentration of 50 µg/mL.

The plant extract inhibited the DPP-IV enzyme activity at the higher concentration of 125 µg/mL, producing about 42% inhibition, and this activity was less than the positive control, diprotin A (Figure 9).

### 3.6. Lipase Inhibition Activity

**Figure 10:**
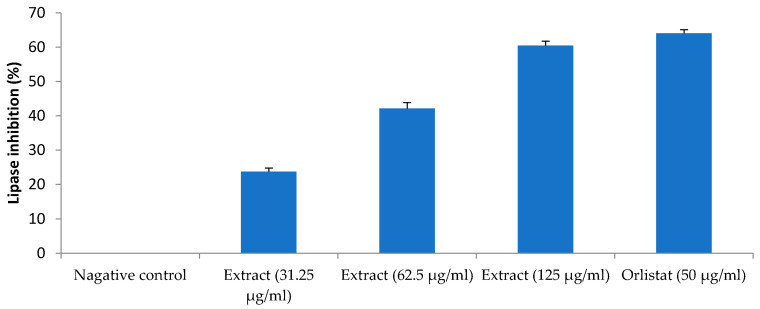
Lipase inhibition activity. All values are mean ± SEM, n = 3. Inhibition of lipase enzyme activity was dose-dependent with maximum inhibition observed with the highest tested concentration of *H. pubescens* (125 µg/mL). Orlistat (50 µg/mL) showed the highest inhibition of the lipase enzyme activity.

As compared to the lipid accumulation and DPP-IV inhibition, the inhibitory effect of *H. pubescens* extract was more profound on the lipase. The extract inhibited the lipase enzyme activity by 23%, 42%, and 60% at concentrations of 31.25 µg/mL, 62.5 µg/mL, and 125 µg/mL, respectively. The standard lipase enzyme inhibitor, orlistat, showed about 64% inhibition of lipase activity at 50 µg/mL (Figure 10).

## 4. Discussion

The present study employed in vivo evaluations in rats to determine the potential effect of *H. pubescens* on serum lipid profile, and in vitro assays were used to explore the mechanisms involved in the hypolipidemic and antihyperlipidemic effects of the extract. Since seeds of the plant have been reported earlier by several authors for various pharmacological activities [20], the present study was carried out using methanolic extract of the *H. pubescens* seeds. Analysis of the methanolic extract showed the presence of several important phytoconstituents that included compounds known to have antidiabetic and hypolipidemic effects. Losartan was used to compare the hypolipidemic action of the extract because *H. pubescens* is mainly used as an antidiabetic drug traditionally with probable hypolipidemic benefits. Hence, a less potent hypolipidemic agent such as losartan was used for comparison [21,22].

The *H. pubescens* extract at the higher dose (500 mg/kg, p.o) was highly effective in reducing the serum cholesterol levels in normal animals, but its effect on serum triglyceride levels was relatively less, and no significant change in the beneficial serum HDL-cholesterol levels was observed. The lower dose of the extract (250 mg/kg, p.o) was less effective, indicating a dose-dependent response. These results suggest that *H. pubescens* may be an effective hypolipidemic agent in normal individuals by reducing cholesterol and triglyceride levels. However, this herb may not cause clearance of cholesterol from blood by increasing the levels of beneficial HDL-cholesterol.

A model to induce hyperlipidemia described by Asdaq et al. [15] was used. The high-fat diet consisted of 3% cholesterol and saturated fat (Dalda). Agents that reduce absorption of cholesterol and triglyceride and those that may enhance lipid clearance from the blood are effective in reducing lipid levels in rats fed a high-fat diet. The animals were initially made hyperlipidemic by feeding them a high-fat diet that increased the serum lipid levels. Animals were fed with the same high-fat diet during the treatment. Comparisons were made with the pretreatment hyperlipidemic values with effects observed on Day 7, Day 14, Day 21, and Day 28 in each group. A comparison was also made between the treatment groups and control groups on Day 7, Day 14, Day 21, and Day 28. These comparisons showed the effects of treatment on serum lipid levels when the high-fat diet was continued, and it also indicated whether drug/extract treatments are better than vehicle treatment. This method may show the effect on agents in individuals who are hyperlipidemic and continue to consume high-fat diets during treatment. In rats fed with a high-fat diet continuously during the treatment, varying effects on cholesterol and triglycerides were observed. The cholesterol levels were significantly reduced as compared to pretreatment levels, while the triglycerides levels continued to rise, though the rise was less as compared to high-fat fed control animals. The rise in triglycerides despite the treatment may be due to the high amount of saturated fat in the diet. The treatments, however, failed to reduce the triglyceride levels as compared to pretreatment values; they were effective in significantly attenuating the rise in serum triglycerides levels as compared to high-fat diet control animals from day 14 onward, indicating their antihyperlipidemic actions. The effects of serum HDL-cholesterol were inconclusive.

To further explore the mechanism involved in the hypolipidemic effect of *H. pubescens*, in vitro studies were carried out. The lipid accumulation assay in 3T3L1 preadipocytes was carried out to determine the contribution of lipid accumulation inside the preadipocyte cells that might have contributed to the hypolipidemic effect of *H. pubescens* extract. The extract did not show a potent effect in increasing the lipid accumulation inside the adipocytes, but it significantly decreased lipid accumulation at the highest tested concentration of 125 µg/mL. Drugs that decrease lipid accumulation within the adipocytes are known to have potential antiobesity activity [23,24]. Rosiglitazone is known to increase the accumulation of lipids in the 3T3L1 preadipocytes [25]. Accumulation of lipids inside the preadipocytes matures them to adipocytes [26]. These results suggest that *H. pubescens* extract not only decreases the serum lipid levels in rats but may also decrease lipid deposition in the preadipocytes, decreasing adipocytes in the body. Further investigation of the antiobesity may provide more insight into the potential of *H. pubescens* as an antiobesity drug.

The dipeptidyl peptidase-4 (DPP-IV) inhibitors that are among the commonly used antidiabetic drugs are known to have a beneficial effect on the lipid profile [27,28]. Diabetic patients have altered lipid levels, and agents that can reduce both blood glucose levels and regulate blood lipid levels will have dual beneficial action. Hence, many of the currently used antidiabetic agents have been reported for hypolipidemic effect [29,30]. As mentioned earlier, *H. pubescens* has been reported earlier for antidiabetic actions [10]. The results showed that *H. pubescens* has a very weak DPP-IV inhibitory effect that might not alter the lipid profile significantly through this mechanism.

The enzyme lipase is secreted by the pancreas, and it helps in the absorption of lipids from the intestine. Inhibition of lipase enzyme activity reduces fat absorption, leading to a decrease in serum lipid levels. Lipase inhibitors such as orlistat are used in the treatment of obesity [31]. The methanolic extract of *H. pubescens* showed good inhibition of lipase enzyme activity in vitro. The reduction in serum triglycerides levels in vivo could be at least in part due to the lipase inhibition activity. *H. pubescens* extract was relatively more effective in reducing serum triglycerides in high-fat fed rats as compared to its effect in rats that received a normal diet. In fact, the high dose of *H. pubescens* extract (500 mg/kg, p.o) was almost as effective as losartan in reducing serum triglyceride levels in high-fat fed rats, suggesting the contribution of lipase inhibition activity mechanism to its overall hypolipidemic and antihyperlipidemic effects.

Some of the constituents identified in the LC-MS analysis are reported to influence lipid levels in the body. Farnesol, one of the reported constituents in *H. pubescens* seeds, is known to reduce lipid accumulation in hepatic cells in vitro [32]. Another constituent, chlorogenic acid, is a known hypolipidemic drug that has been reported to reduce serum lipids in high-fat-fed mice [33]. Similarly, acacetin, a flavones present in the seeds, protects against the accumulation of lipids in liver [34]. Phytochemical analysis of the seed extract to reveal different chemical constituents and their effect on lipids may provide more insight into the active constituents that contributed to the hypolipidemic and antihyperlipidemic actions. This may also lead to the development of new molecules as potential hypolipidemic and antiobesity agents. The present study was carried out using methanolic extract, which is known to contain mainly polar constituents [35]. The above-mentioned constituents are also polar in nature. There are no reports on the type and effects of nonpolar constituents of the seeds on the lipid profile. A detailed study about the effect of seed extracts using nonpolar solvents may reveal the potential nonpolar hypolipidemic agents.

As mentioned earlier, *H. pubescens* possesses good antidiabetic activity [10]. It is well known many diabetic patients suffer from hyperlipidemia caused by overproduction of triglyceride-rich lipoproteins and a decrease in beneficial HDL levels [36]. The present study was conducted using nondiabetic animals that received either a normal diet or high-fat diet. Studying the hypolipidemic effect of *H. pubescens* in diabetic animals may provide useful information about the effectiveness of this herb in altering serum lipid levels when used in the treatment of diabetes. The results obtained on serum triglyceride levels and in vitro inhibition of lipoprotein lipase suggest that *H. pubescens* extract may reduce triglyceride levels in diabetic individuals. In contrast, the results obtained with the effect on HDL levels suggest that *H. pubescens* may not reverse the decreased HDL levels in diabetic animals, as there was no effect on serum HDL levels in both normal animals and high-fat-diet fed animals.

This study has a few limitations that need to be addressed with further investigation. Studies on isolated compounds for hypolipidemic and antihyperplipidemic effects by both in vivo and in vitro methods are required to identify the constituents(s) responsible for the effect that was observed in the current study. Many of the hypolipidemic herbs are known to prevent development of atherosclerosis through multiple mechanisms, among which the most prominent effect is antioxidant action. Many of the constituents identified in the current study are known to possess antioxidant effects. Evaluation of the extract and its components for antioxidant and antiatherosclerotic effects may provide more insight into its therapeutic potential in the prevention and treatment of atherosclerosis. Only a few mechanisms for hypolipidemic action were studied in the present work. Other mechanisms of action such as HMG-CoA reductase inhibition, action of bile secretion, and sequestration must be studied.

## 5. Conclusions

The methanolic extract of *H. pubescens* seeds reduced serum cholesterol and serum triglyceride levels in rats with no significant action on serum HDL-cholesterol levels. The effects were similar in rats that either received a normal diet or those fed a high-fat diet. *H. pubescens* seed extract was highly effective in reducing lipase enzyme activity in vitro but had a modest effect on the inhibition of lipid accumulation in preadipocytes and inhibition of DPP-IV. The reduction in lipase enzyme activity might have contributed at least partly to the in vivo hypolipidemic and antihyperlipidemic actions of the seed extract.

## Figures and Tables

**Table 1:** Contents of rat animal feed.

Contents	Percentage
Crude Protein	18.18%
Crude Fat	3.20%
Crude Fibers	5.00%
Total Ash	5.45%
Calcium	1.26%
Salts	0.5%
Phosphorus	0.54%
Vitamin A	20 IU/mg
Vitamin B	20 IU/mg
Vitamin E	2.2 IU/mg

**Table 2:** List of compounds identified by LC-MS.

Number	Compound Name	Formula	Ion	Observed Mass
1.	4-(Methylsulfinyl)butylglucosinolate	C_20_H_41_NO_4_	Positive	359.303
2.	7-Hydroxy-4-methylcoumarin	C_10_H_8_O_3_	Positive	176.047
3.	Canthaxanthin	C_20_H_41_NO_4_	Positive	359.303
4.	Chlorogenic acid Hemihydrate	C_16_H_18_O_9_	Positive	354.095
5.	Cystathionine	C_20_H_41_NO_4_	Positive	359.303
6.	DL-Dihydrozeatin	C_13_H_20_O_3_	Positive	224.141
7.	Esculin sesquihydrate	C_16_H_18_O_9_	Positive	354.095
8.	Farnesol (mixture of isomers)	C_13_H_20_O_3_	Positive	224.141
9.	Fusaric acid	C_10_H_13_NO_2_	Positive	179.094
10.	Methyl Jasmonate	C_13_H_20_O_3_	Positive	224.141
11.	N-Acetyl-Phytosphingosine	C_20_H_41_NO_4_	Positive	359.303
12.	Rape seed mixture glucosinolates	C_23_H_24_O_13_	Positive	347.22
13.	Scoulerin	C_19_H_21_NO_4_	Positive	327.147
14.	Sodium Deoxycholate	C_24_H_40_O_4_	Positive	392.292
15.	1-Myristoyl-2-Hydroxy-sn-Glycero-3-Phosphate (Sodium Salt)	C_17_H_35_O_7_P	Negative	381.2724
16.	2′-Deoxycytidine	C_9_H_13_N_3_O_4_	Negative	227.2955
17.	6-Phosphogluconic acid Barium salt hydrate	C_6_H_13_O_10_P	Negative	279.3961
18.	Acacetin	C_16_H_12_O_5_	Negative	281.3870
19.	alpha-D-Galactose-1-phosphate Dipotassium Salt	C_6_H_13_O_9_P	Negative	255.3365
20.	D-(-)-Quinic acid	C_7_H_12_O_6_	Negative	191.0891
21.	D-Glucosamine-6-phosphate sodium salt	C_6_H_14_NO_8_P	Negative	253.3457
22.	D-Glucuronic acid	C_6_H_10_O_7_	Negative	193.1474
23.	Lignoceric Acid	C_24_H_48_O_2_	Negative	367.2342
24.	Uridine-5′-monophosphate	C_9_H_13_N_2_O_9_P	Negative	325.3563
25.	Xanthosine	C_10_H_12_N_4_O_6_	Negative	283.4454
26.	Xanthosine-5′-monophosphate disodium salt	C_10_H_13_N_4_O_9_P	Negative	367.2679

## Data Availability

Data will be supplied on request from the readers.

## Supplementary Materials

The following supporting information can be downloaded at: https://www.mdpi.com/article/10.3390/life13071435/s1, Figure S1: Mass spectra of suspected molecules.
