# Supplementary material for: Hypolipidemic and Antihyperlipidemic Effects of Holarrhena pubescens Methanolic Extract Is Mediated through Inhibition of Lipase Activity and Lipid Accumulation
Source: Life (Basel). 2023 Jun 24;13(7):1435. doi: 10.3390/life13071435 (PMC10381764; doi:10.3390/life13071435)

Figure S1: Mass spectra of suspected molecules

4-(Methylsulfinyl)butylglucosinolate

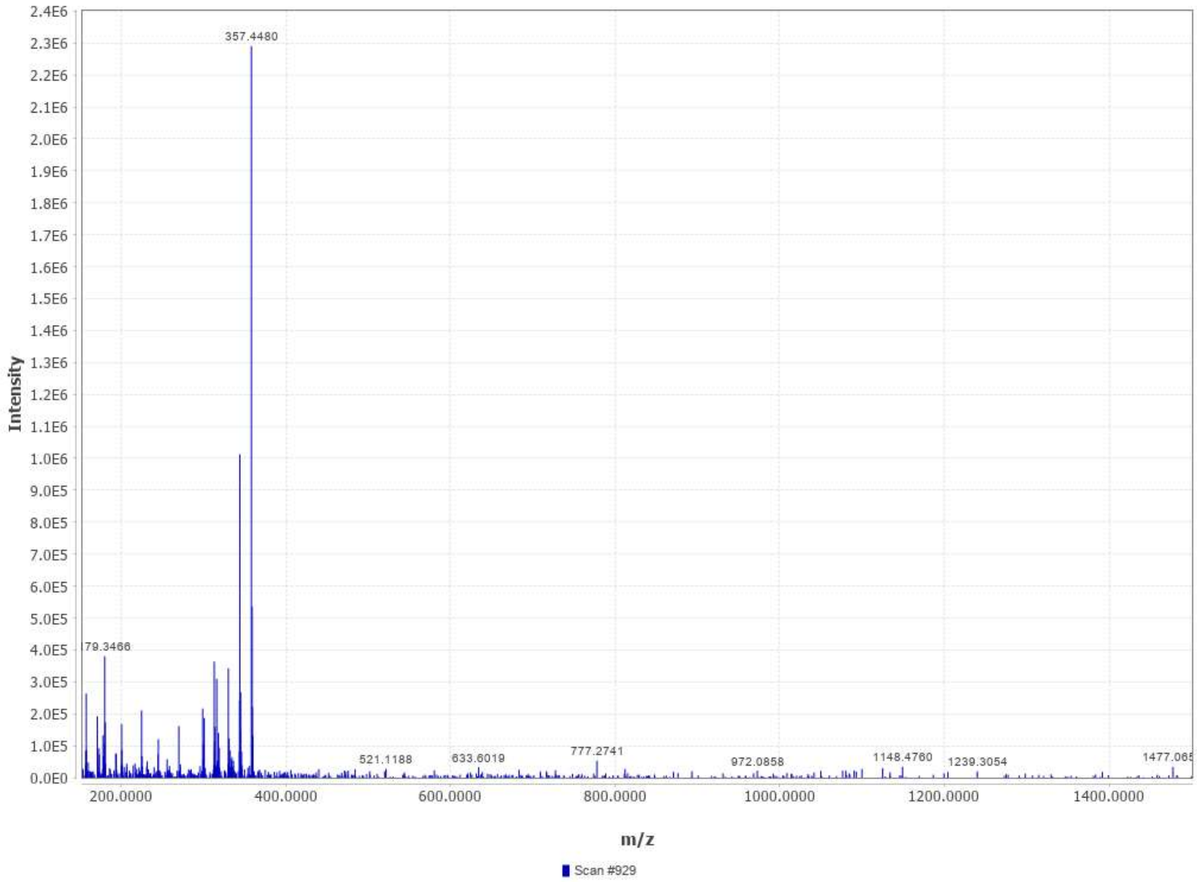

7-Hydroxy-4-methylcoumarin

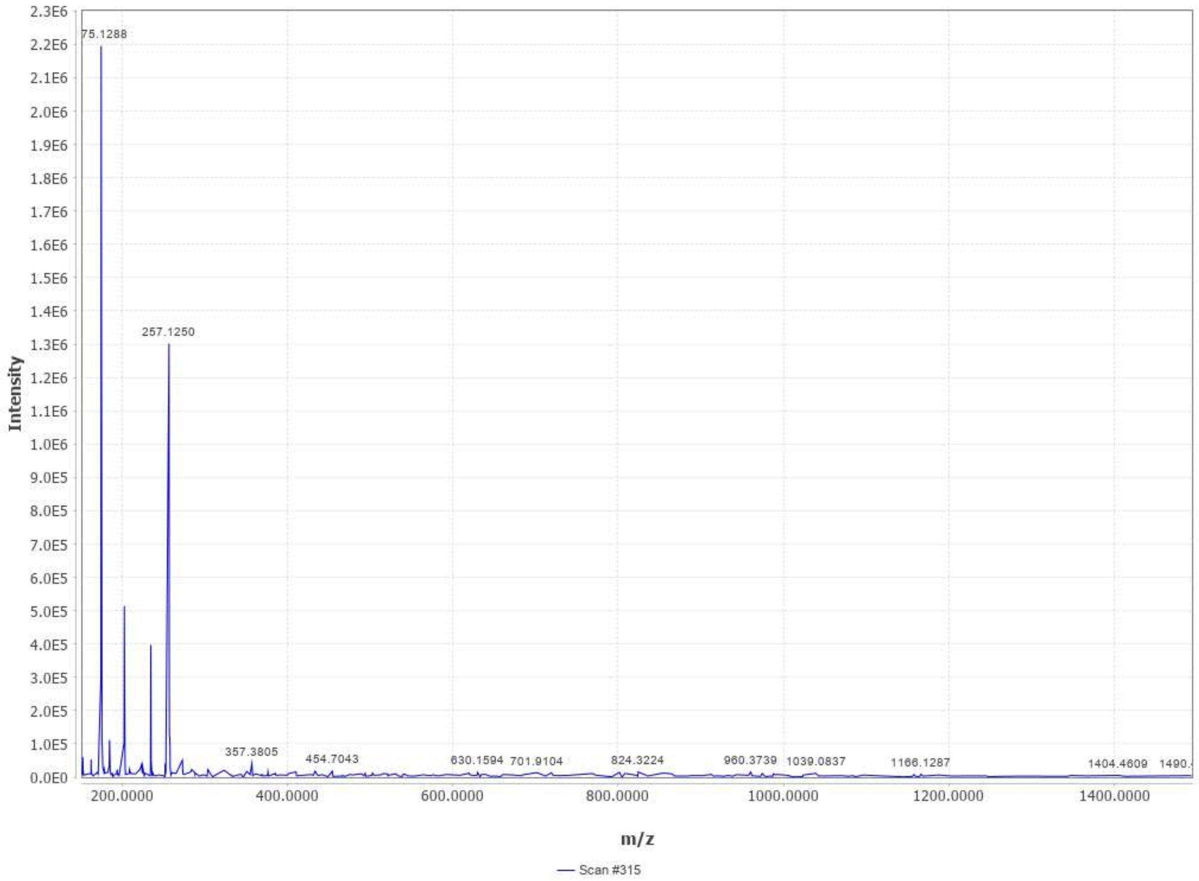

<u>**Canthaxanthin**</u>

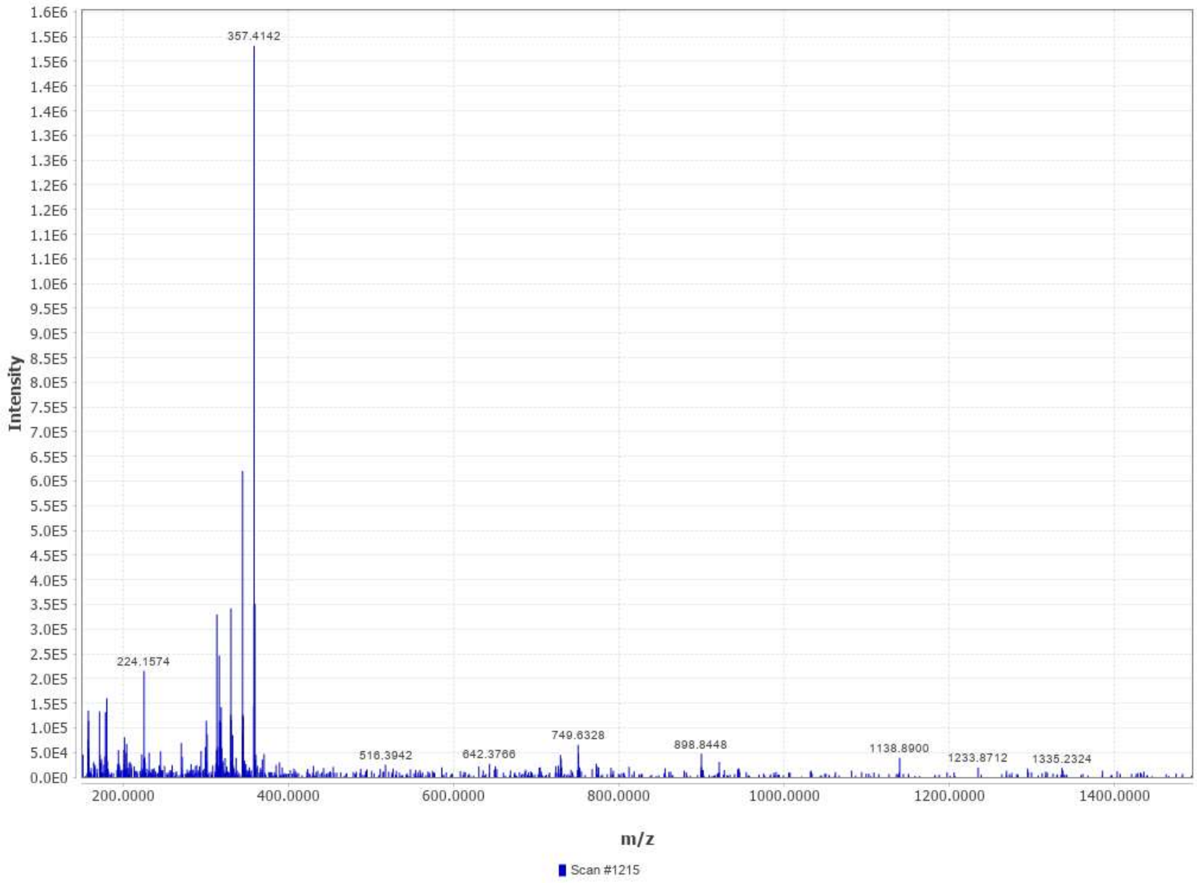

Chlorogenic acid hemihydrate

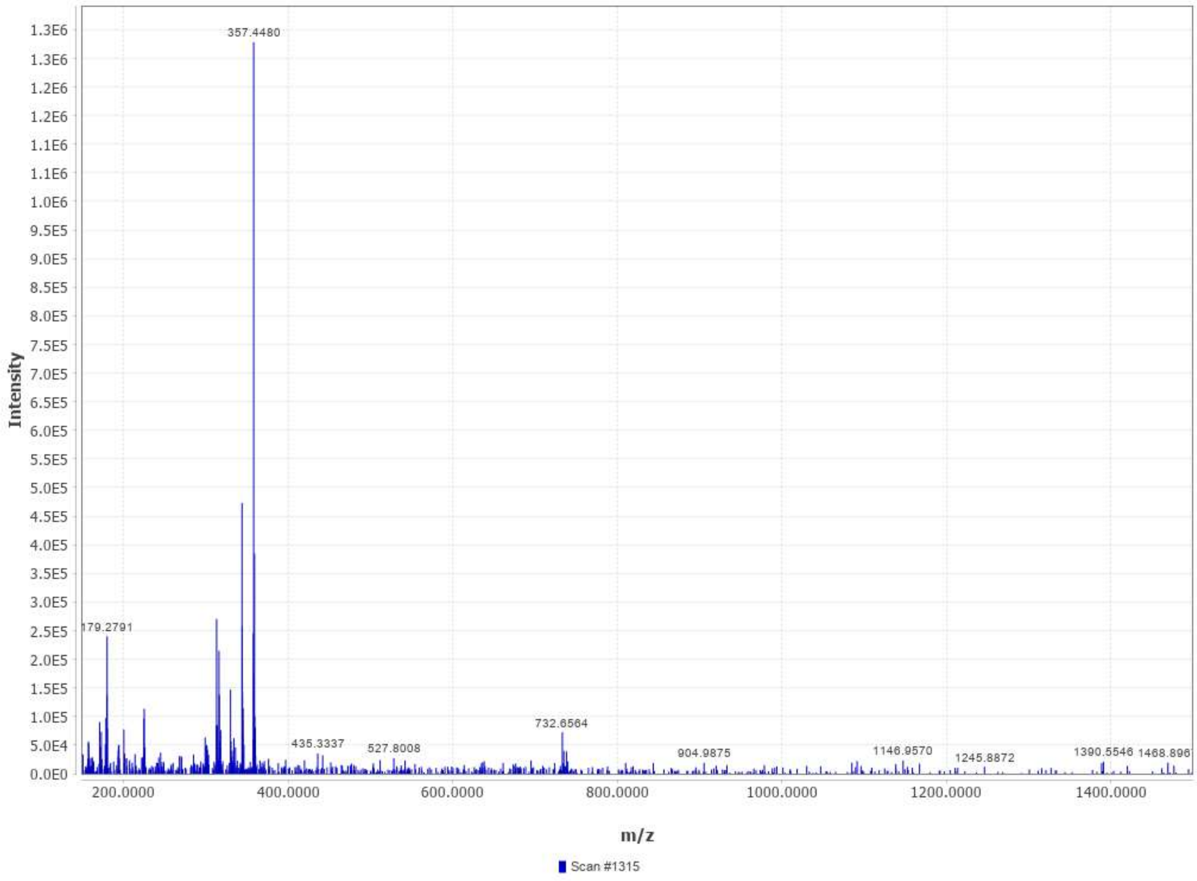

Cystathionine

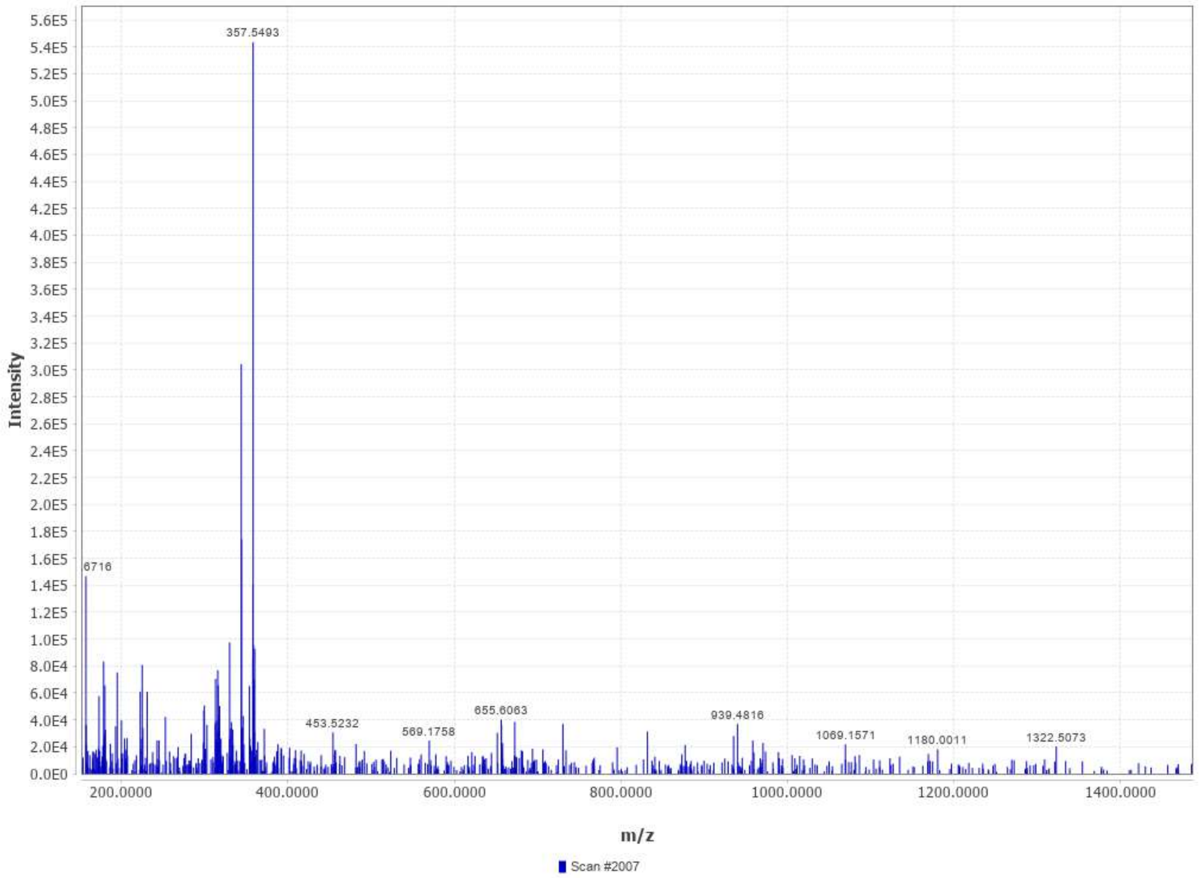

DL-Dihydrozeatin

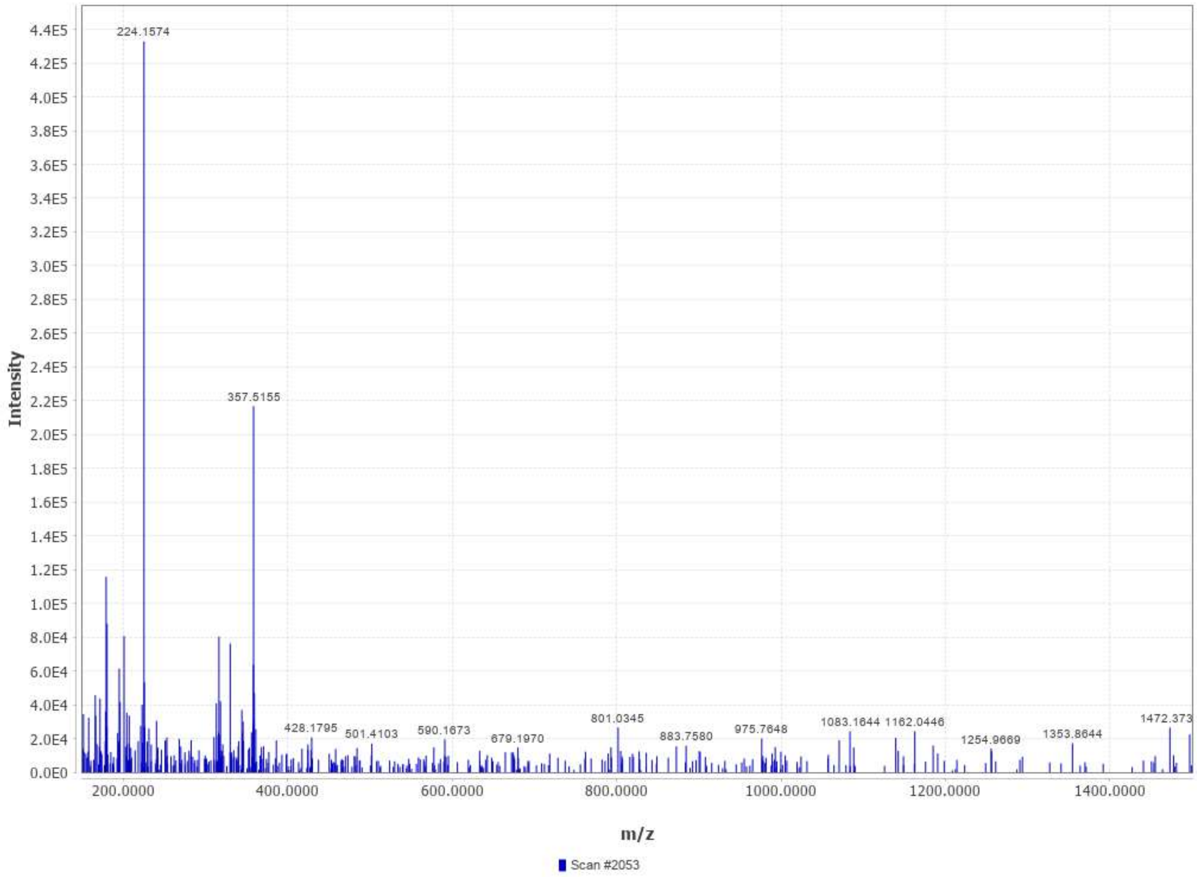

Esculin sesquihydrate

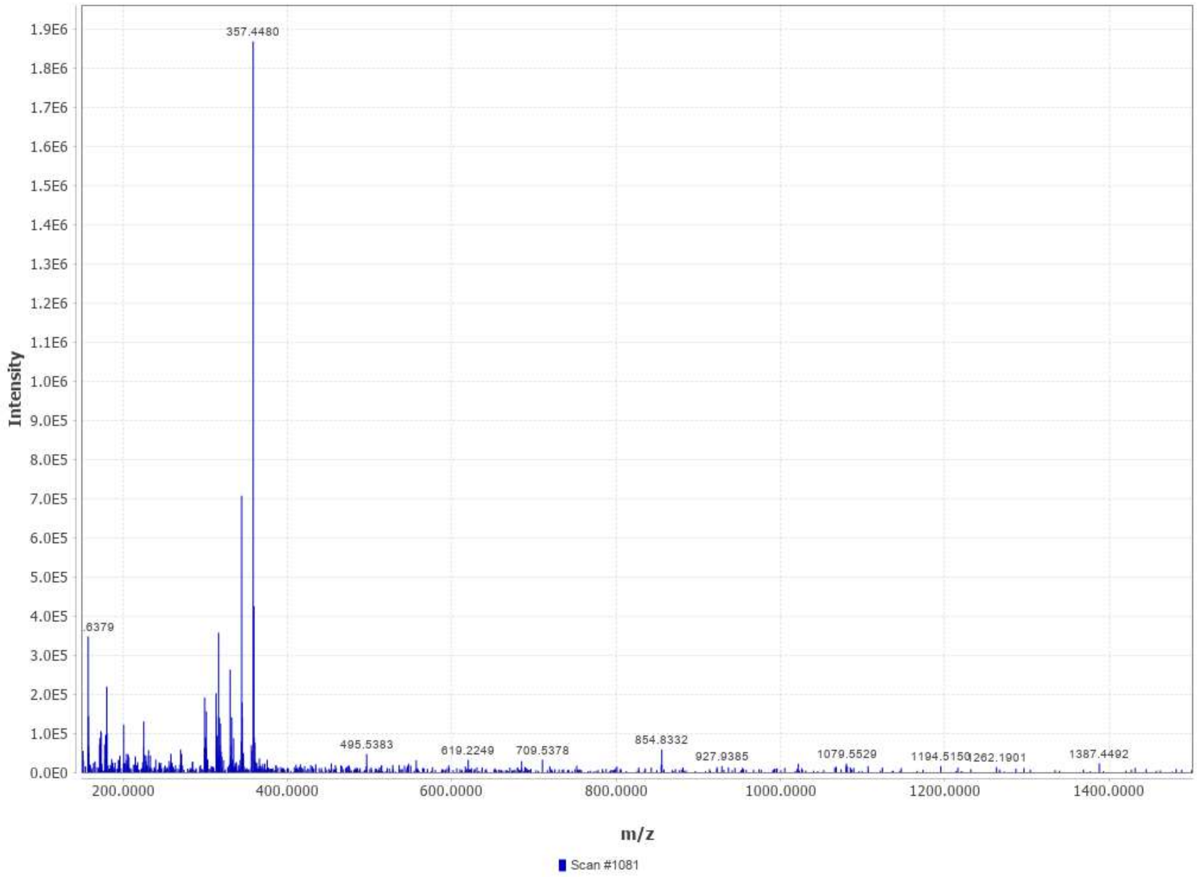

<u>Farnesol (mixture of isomers)</u>

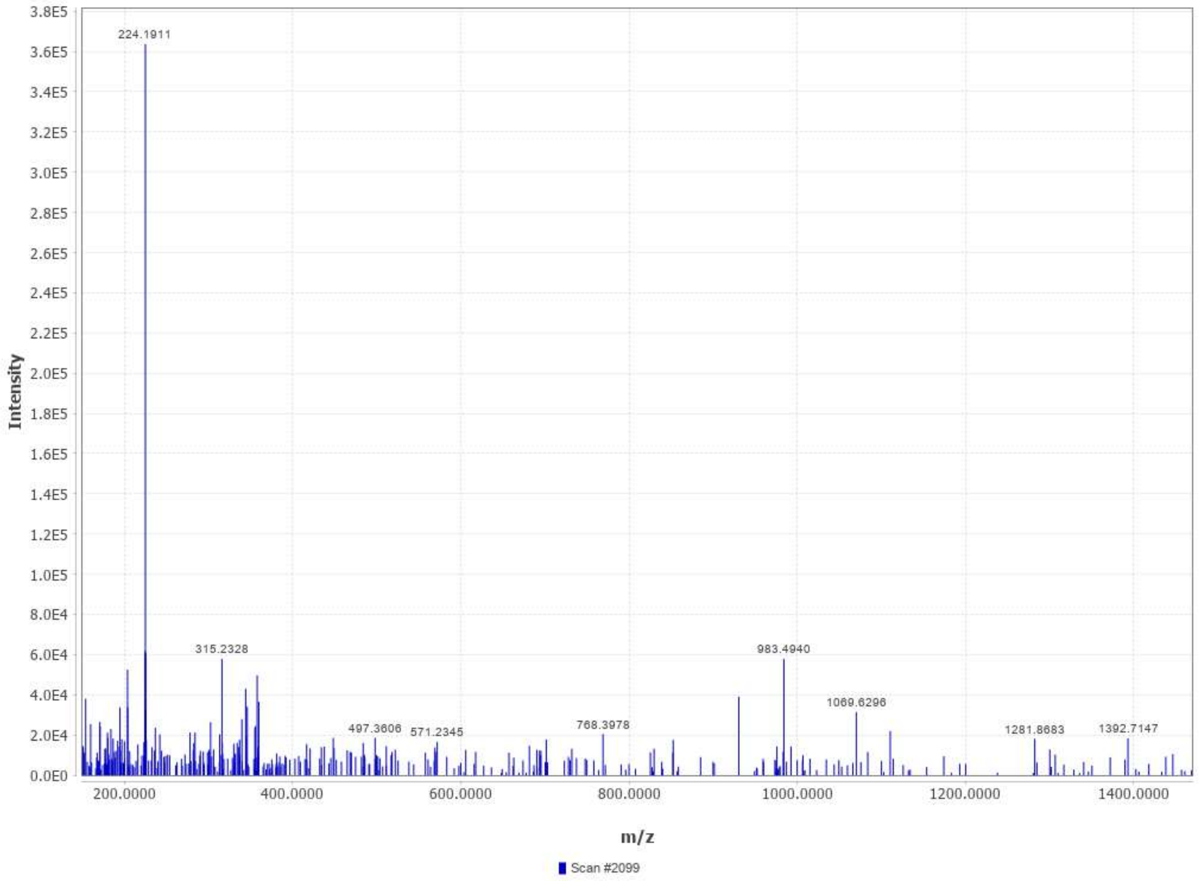

Fusaric acid

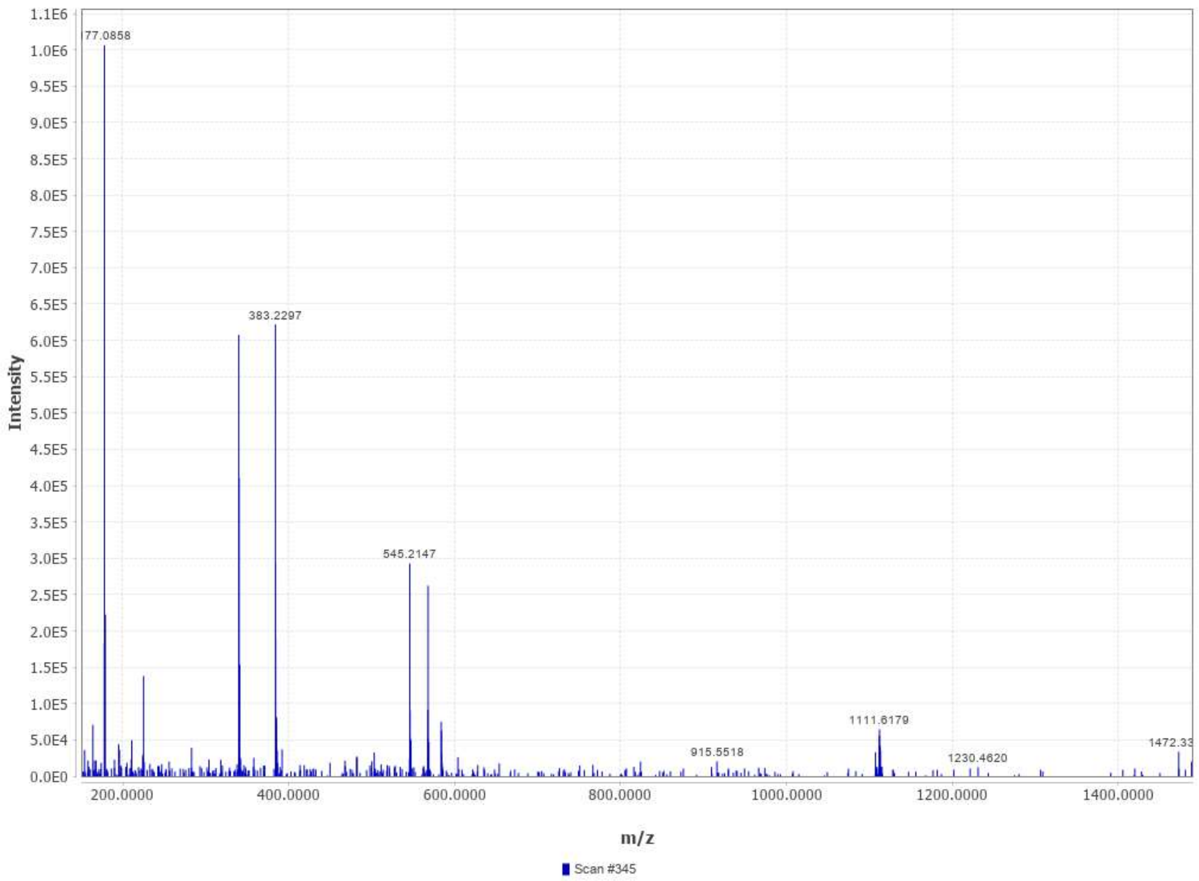

Methyl Jasmonate

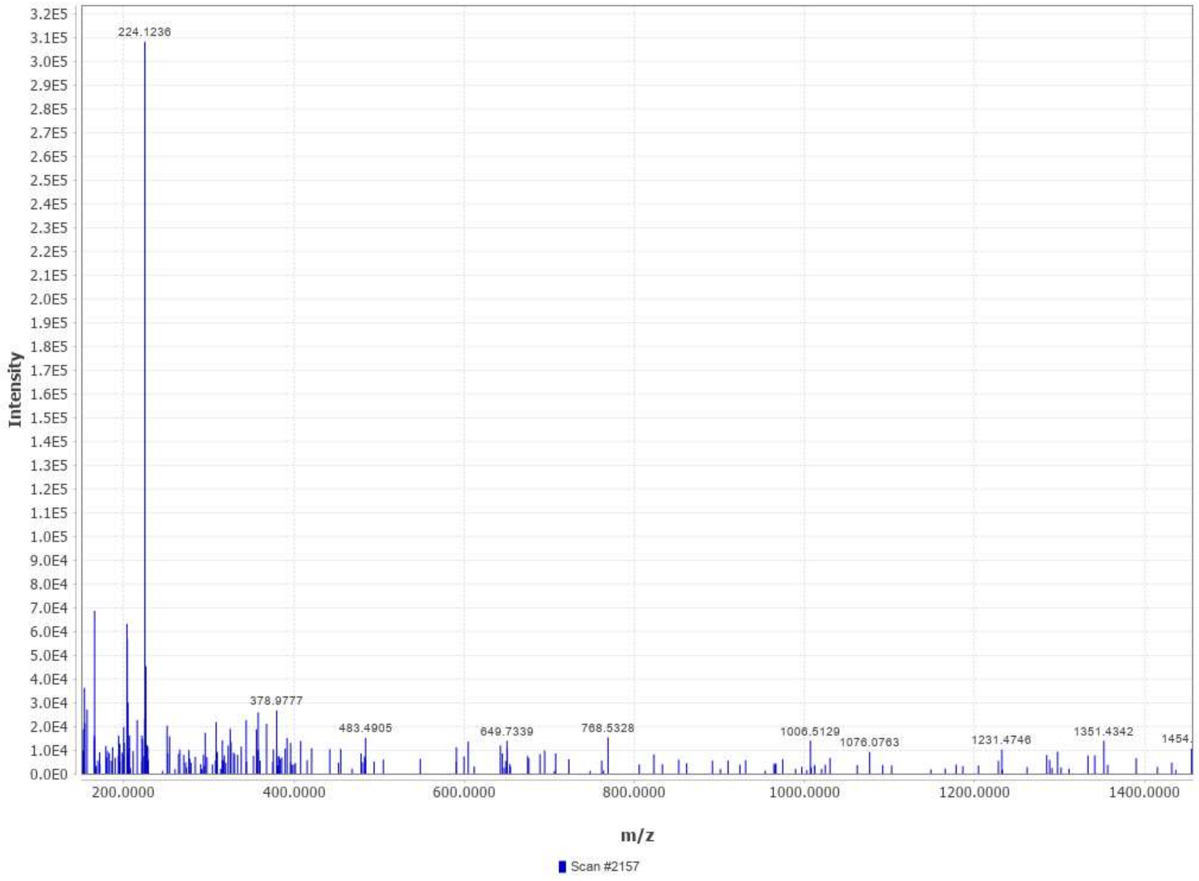

N-Acetyl-Phytosphingosine

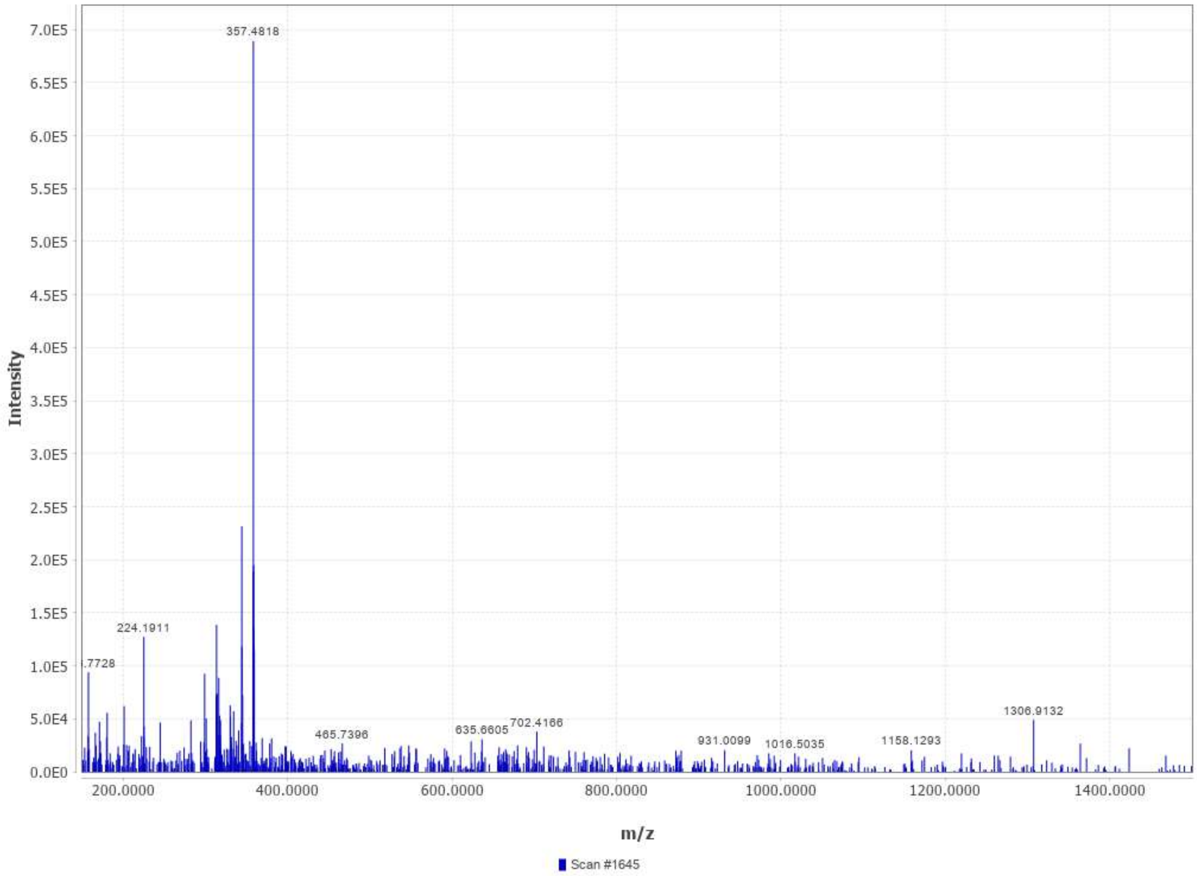

Rape seed mixture glucosinolates

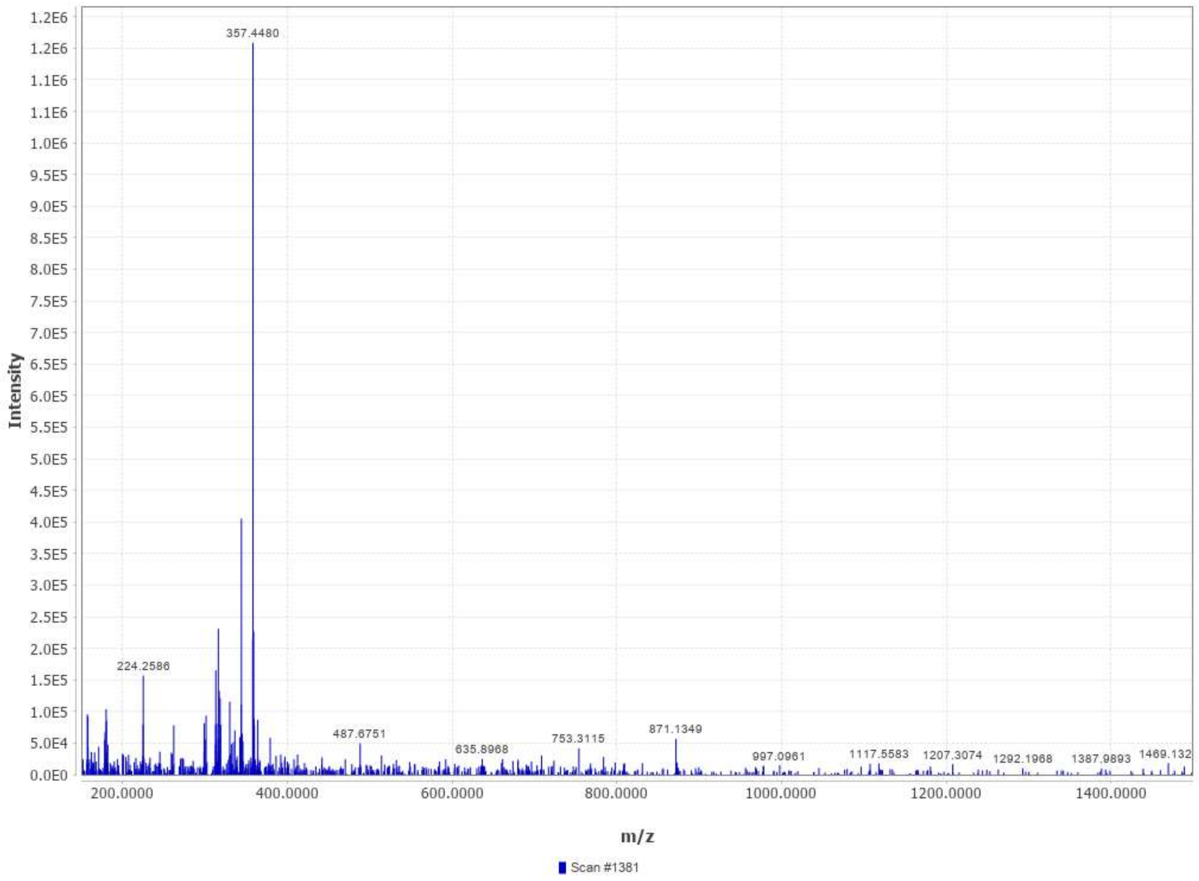

Scoulerin

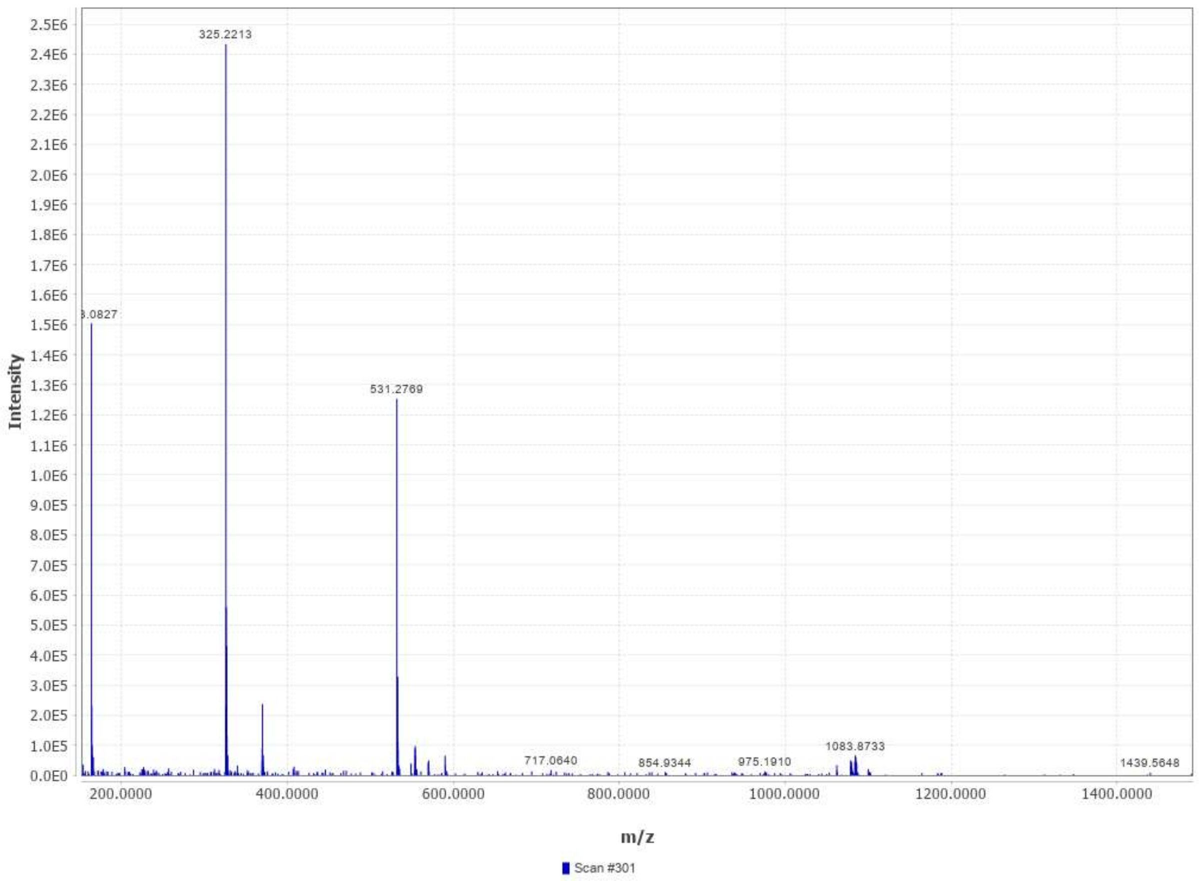

Intensity
m/z
325.2213
3.0827
531.2769
717.0640
854.9344
975.1910
1083.8733
1439.5648
Scan #301

Sodium Deoxycholate

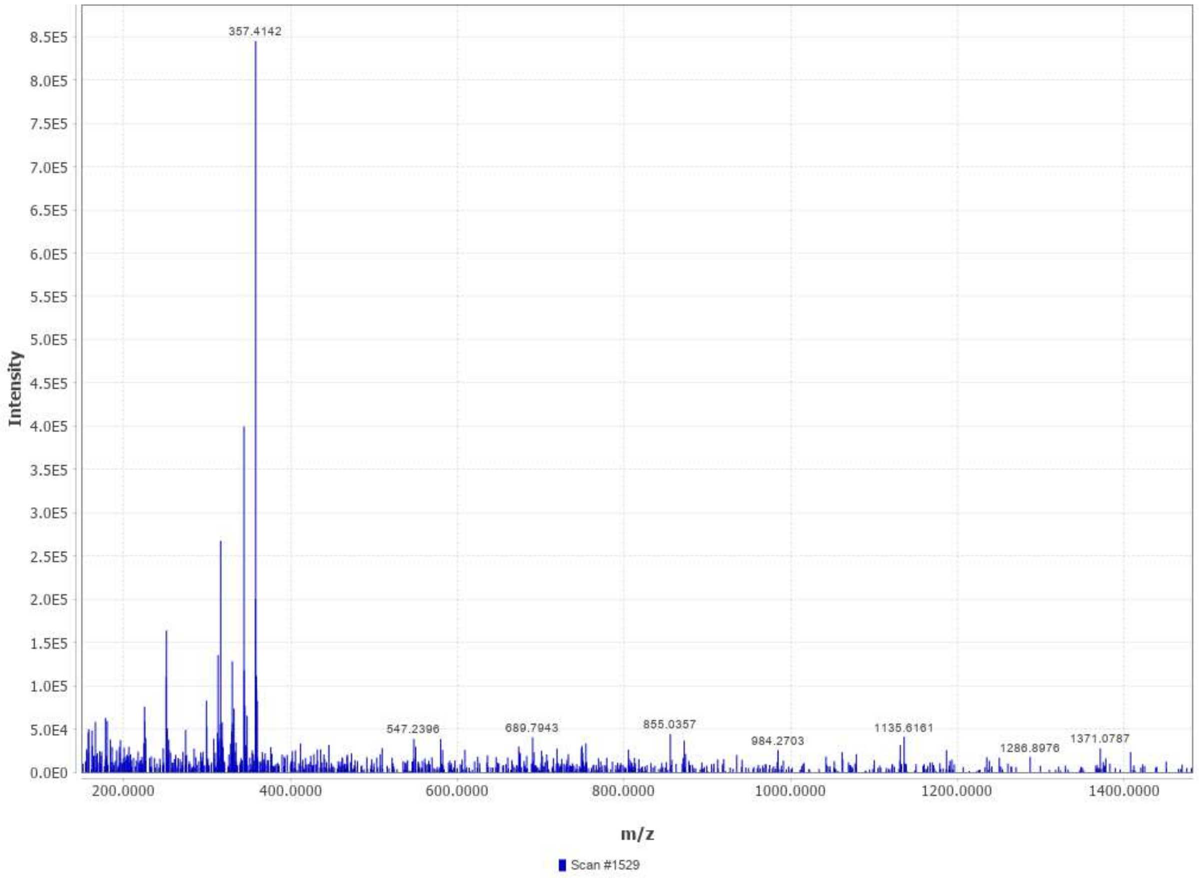

__1-Myristoyl-2-Hydroxy-sn-Glycero-3-Phosphate (Sodium Salt) Sodium Salt__

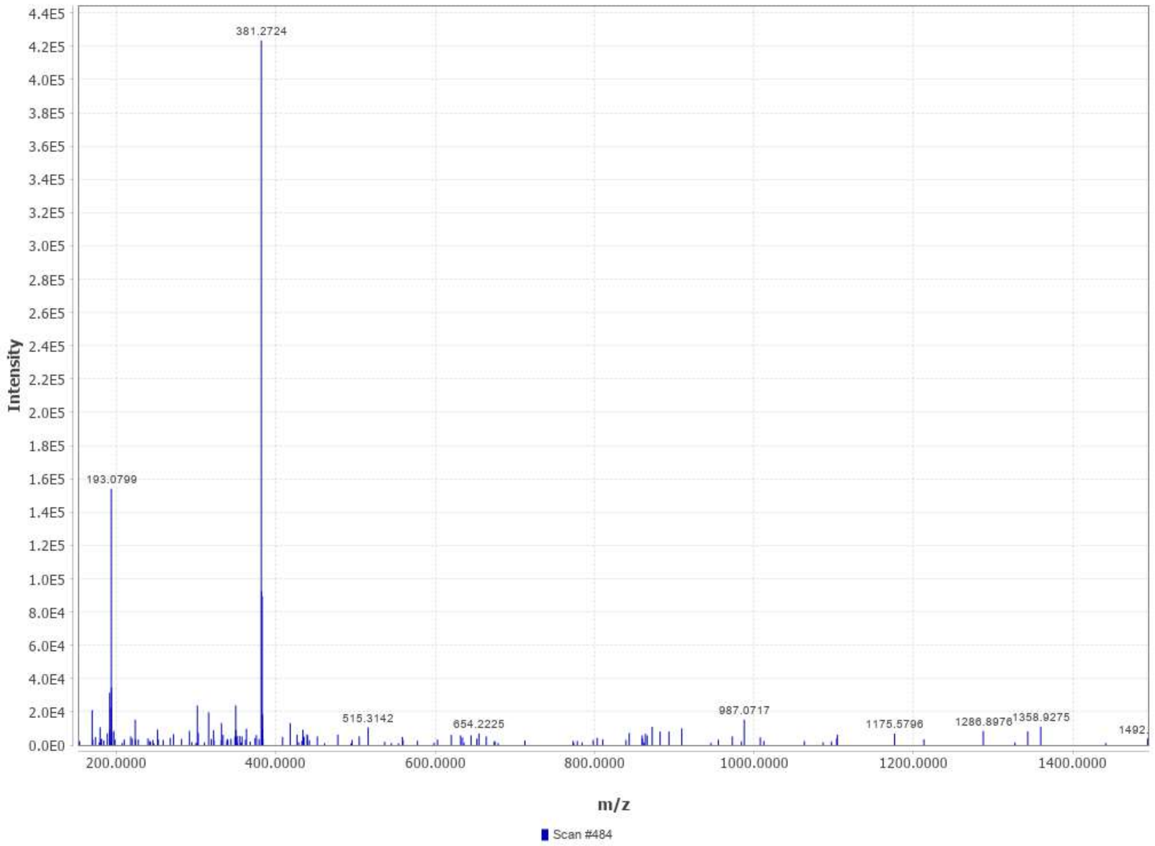

2'-Deoxycytidine

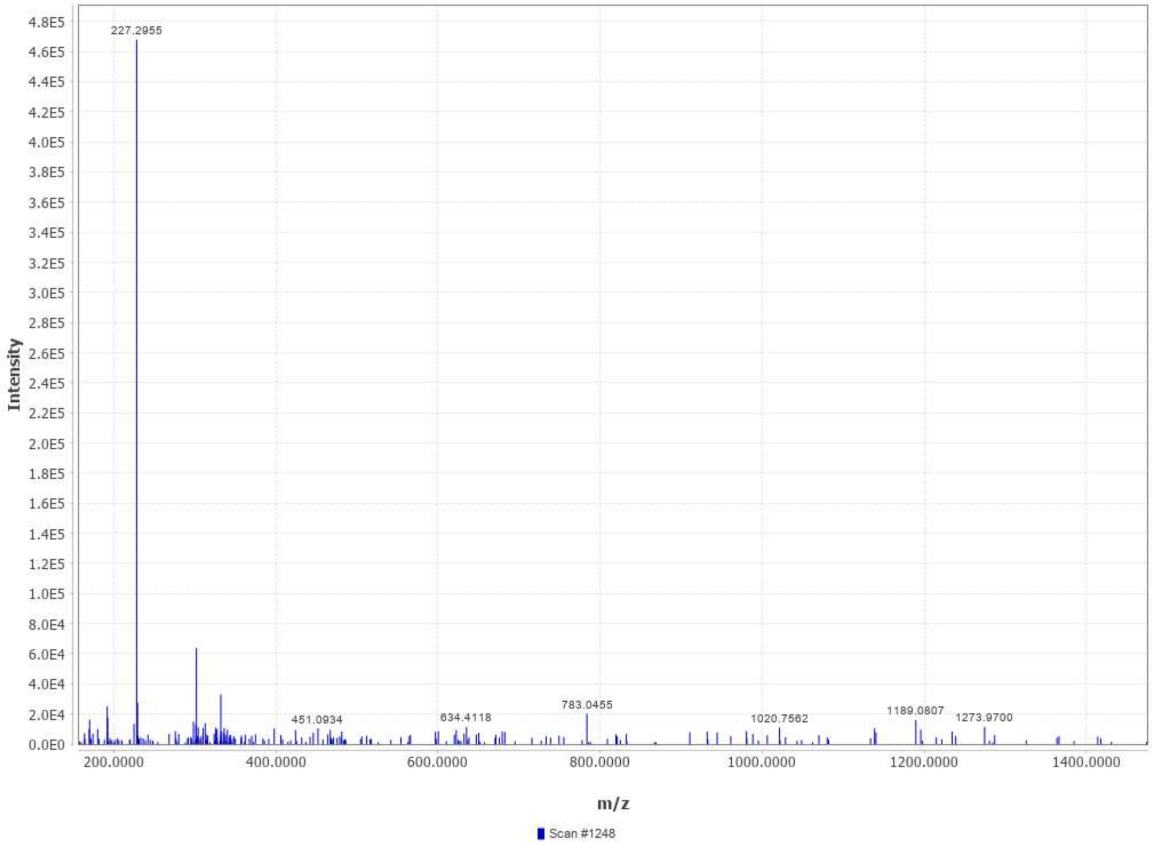

6-Phosphogluconic acid Barium salt hydrate

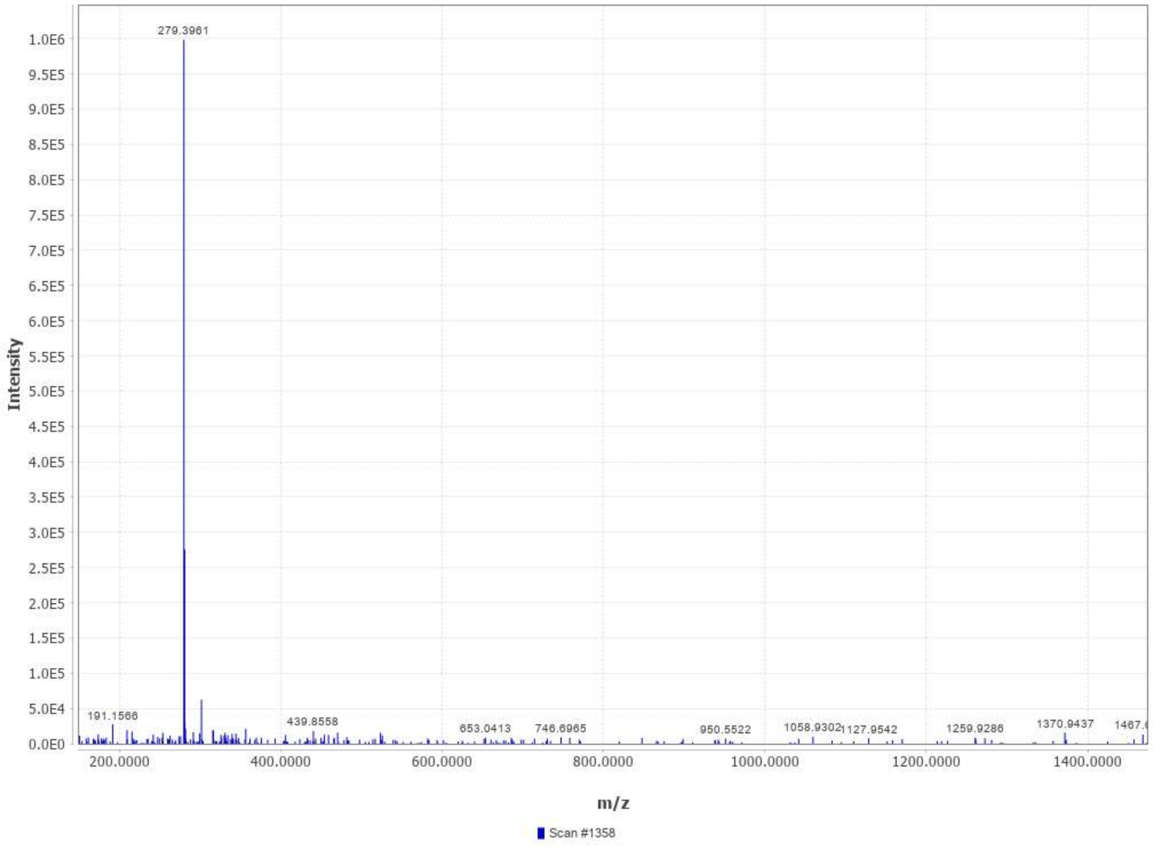

Acacetin

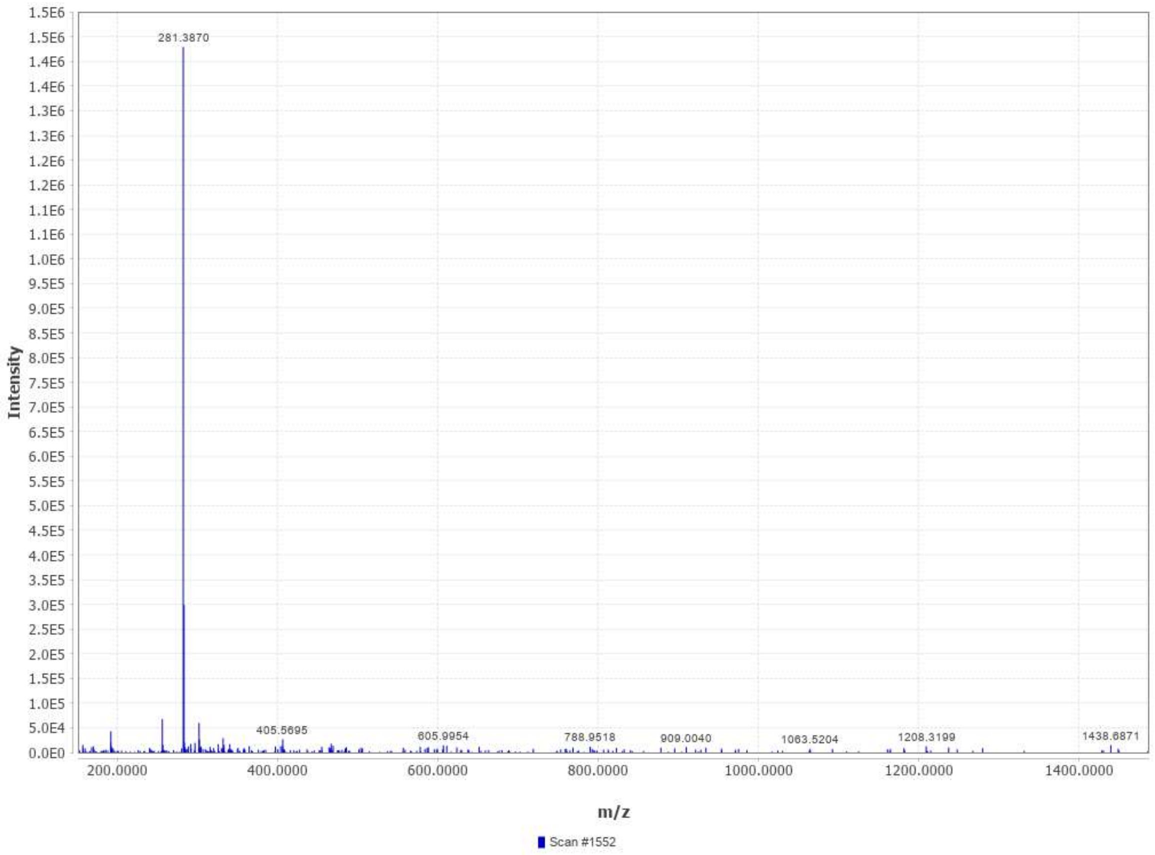
281.3870
405.5695
605.9954
788.9518
909.0040
1063.5204
1208.3199
1438.6871
Intensity
m/z
200.0000
400.0000
600.0000
800.0000
1000.0000
1200.0000
1400.0000
Scan #1552

alpha-D-Galactose-1-phosphate Dipotassium Salt

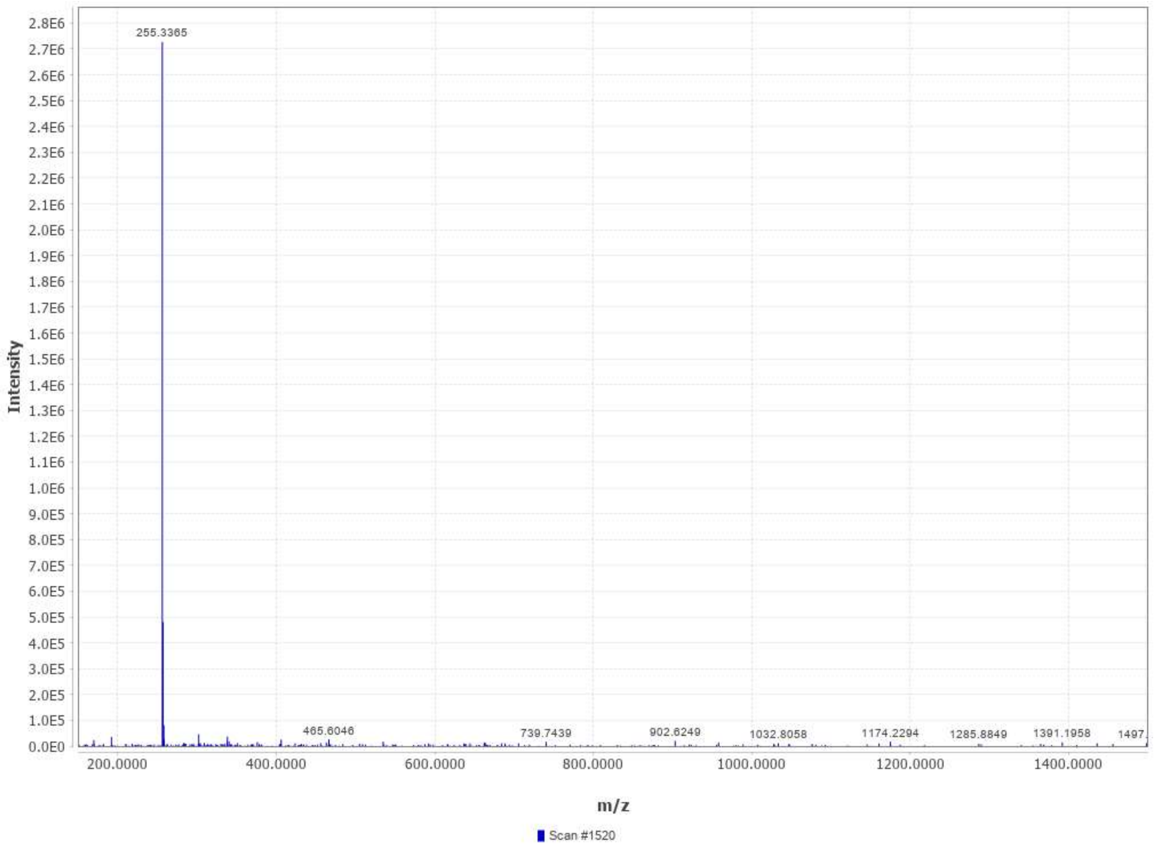

D-(-)-Quinic acid

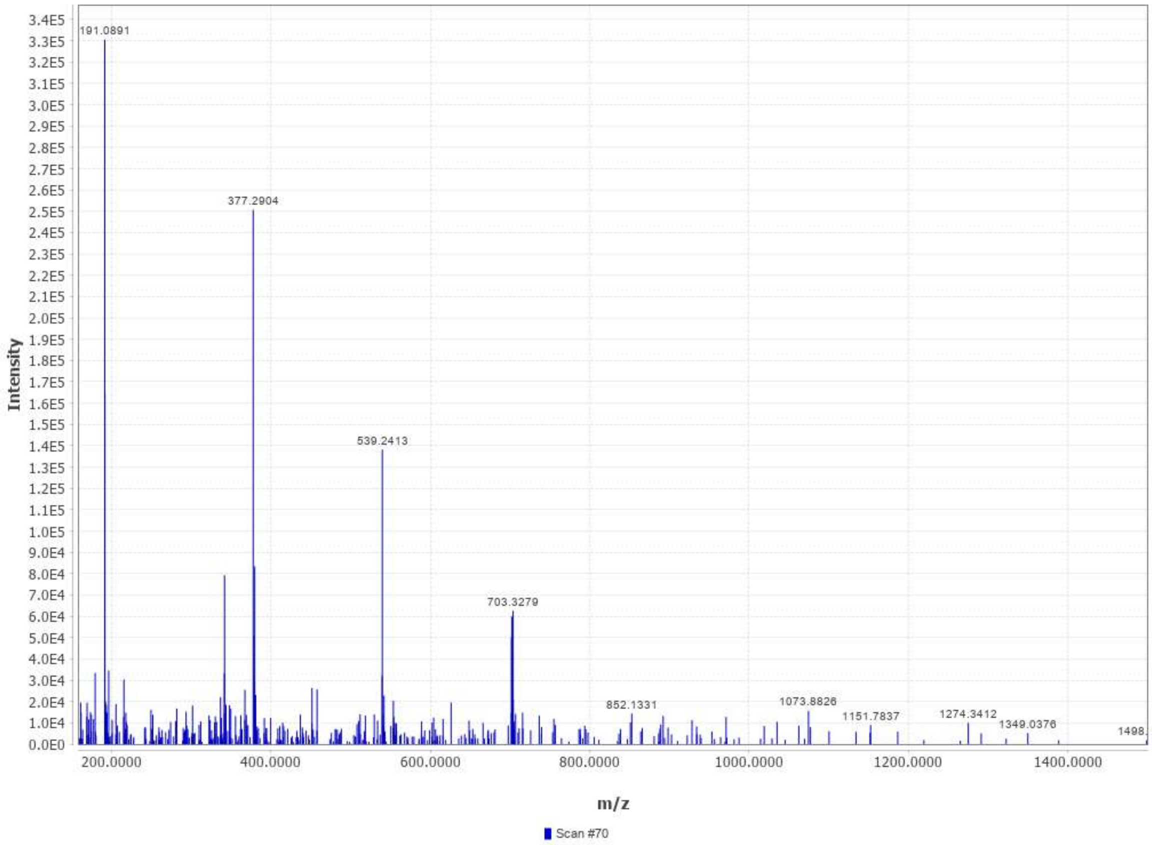

D-Glucosamine-6-phosphate sodium salt

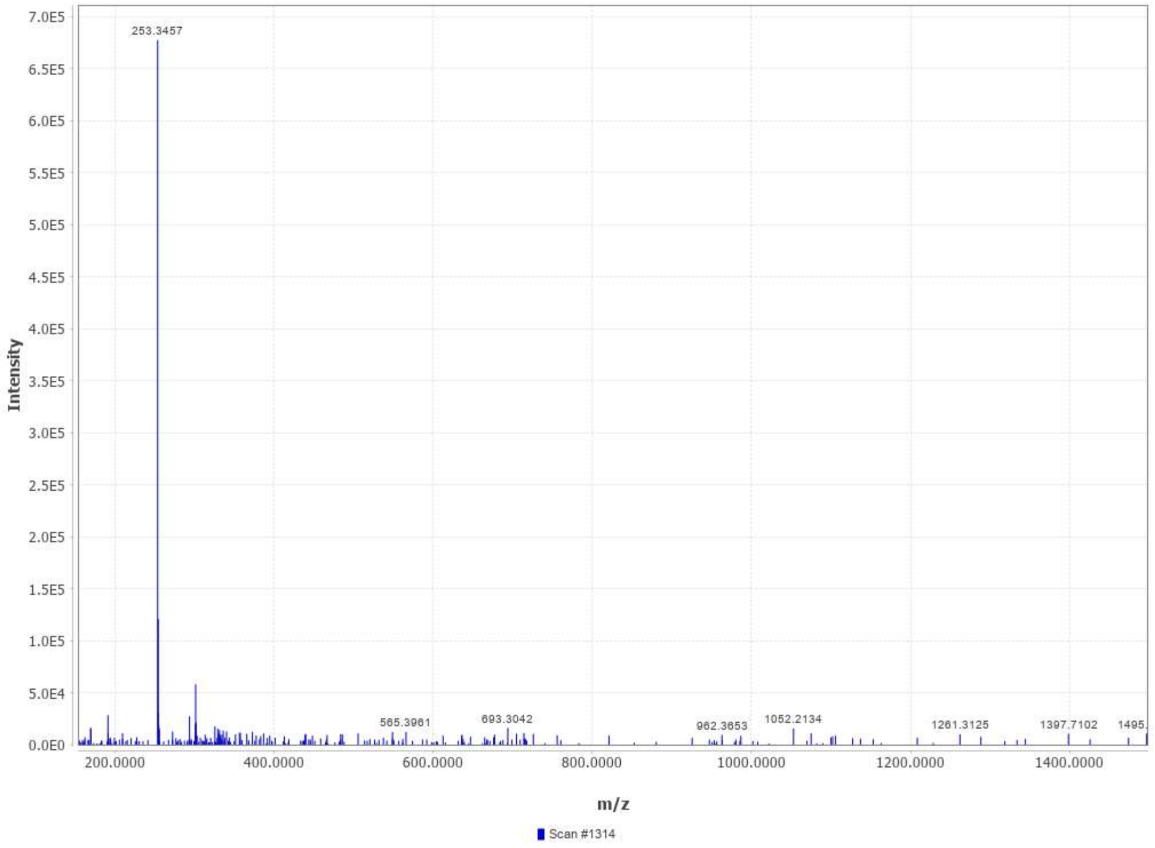

D-Glucuronic acid

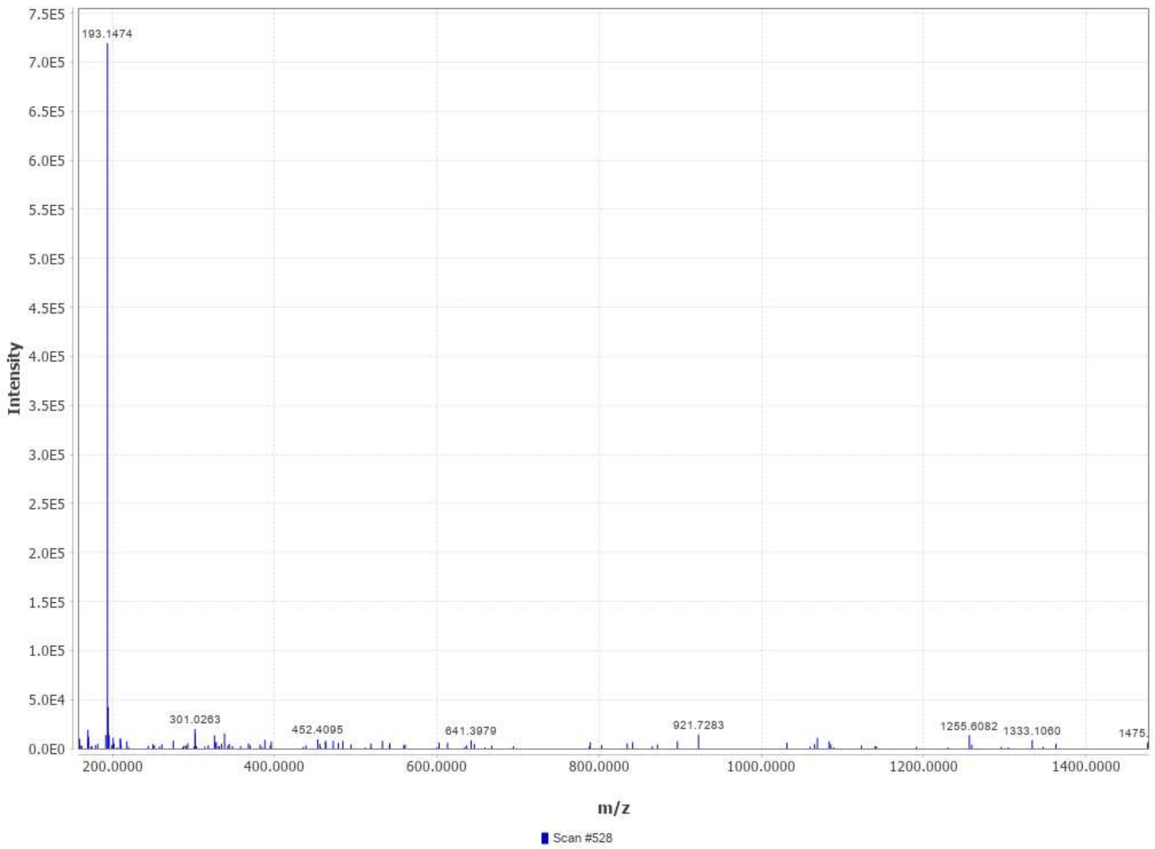

Lignoceric Acid

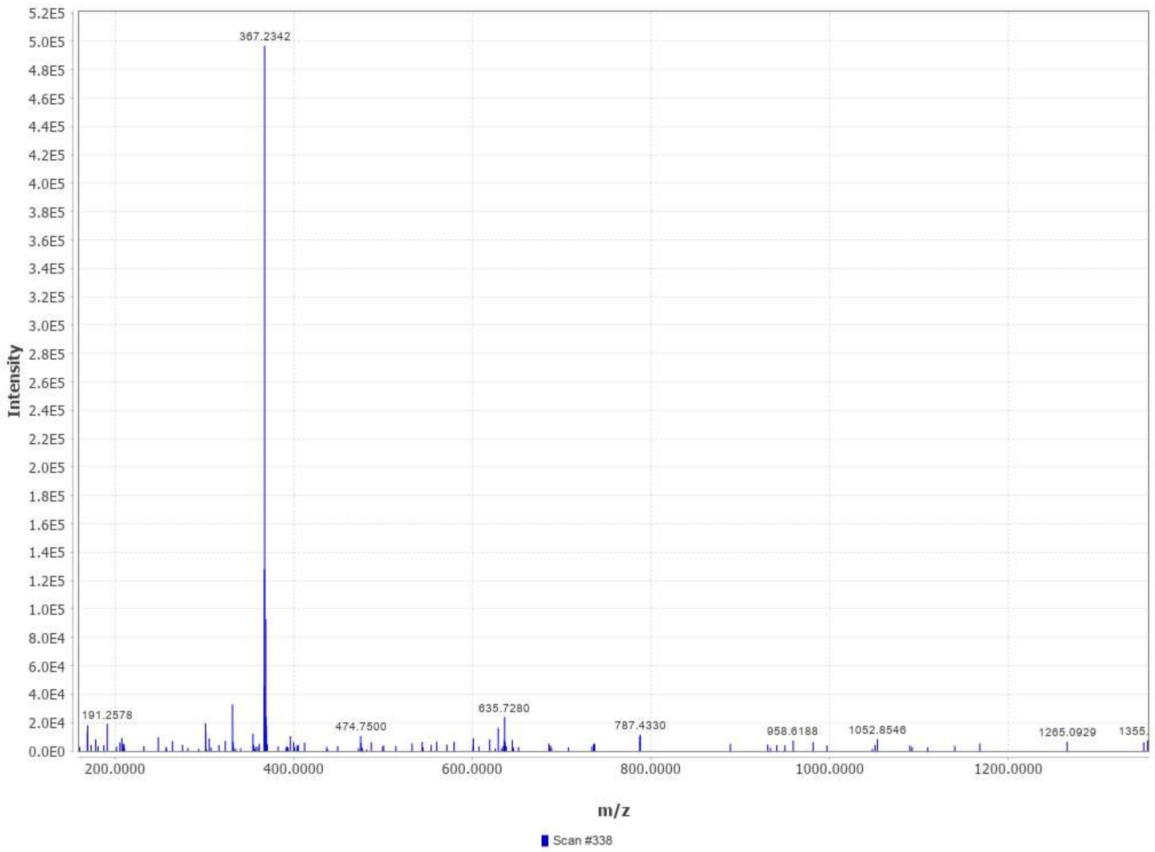

Uridine-5'-monophosphate

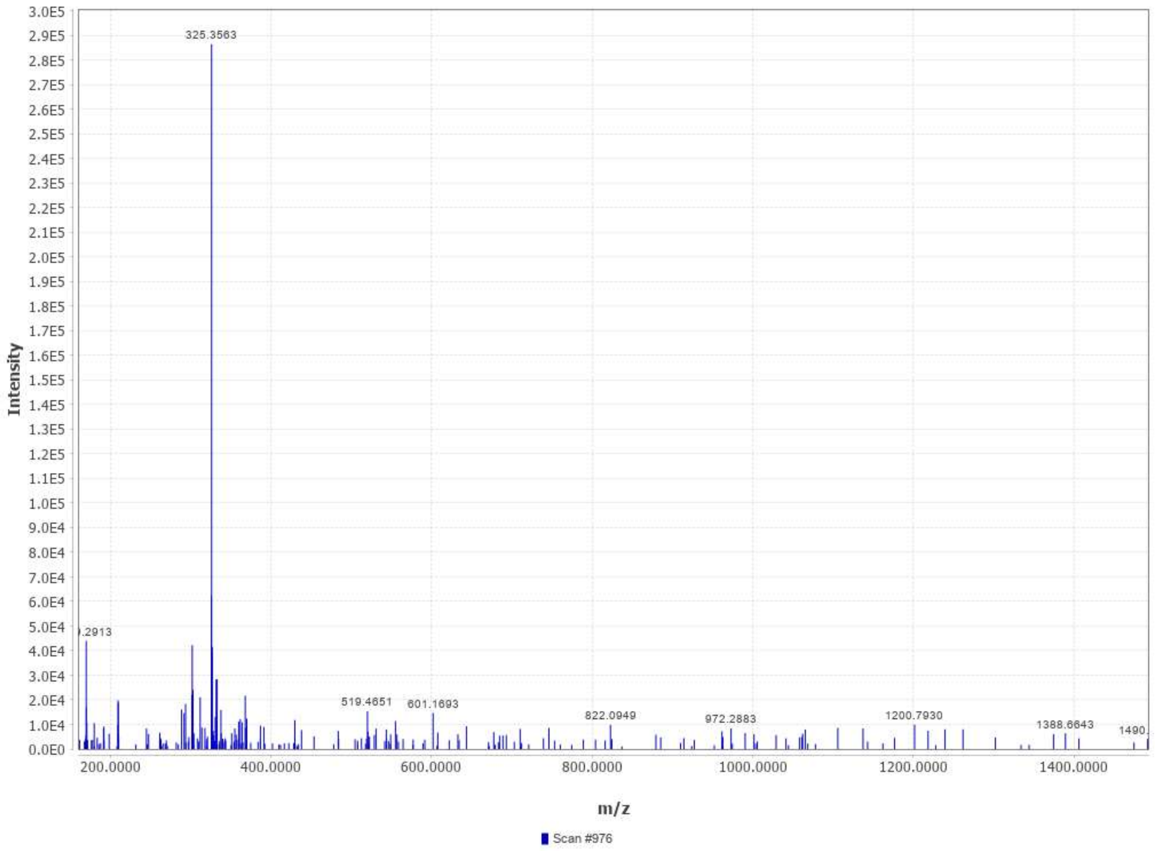

<u>Xanthosine</u>

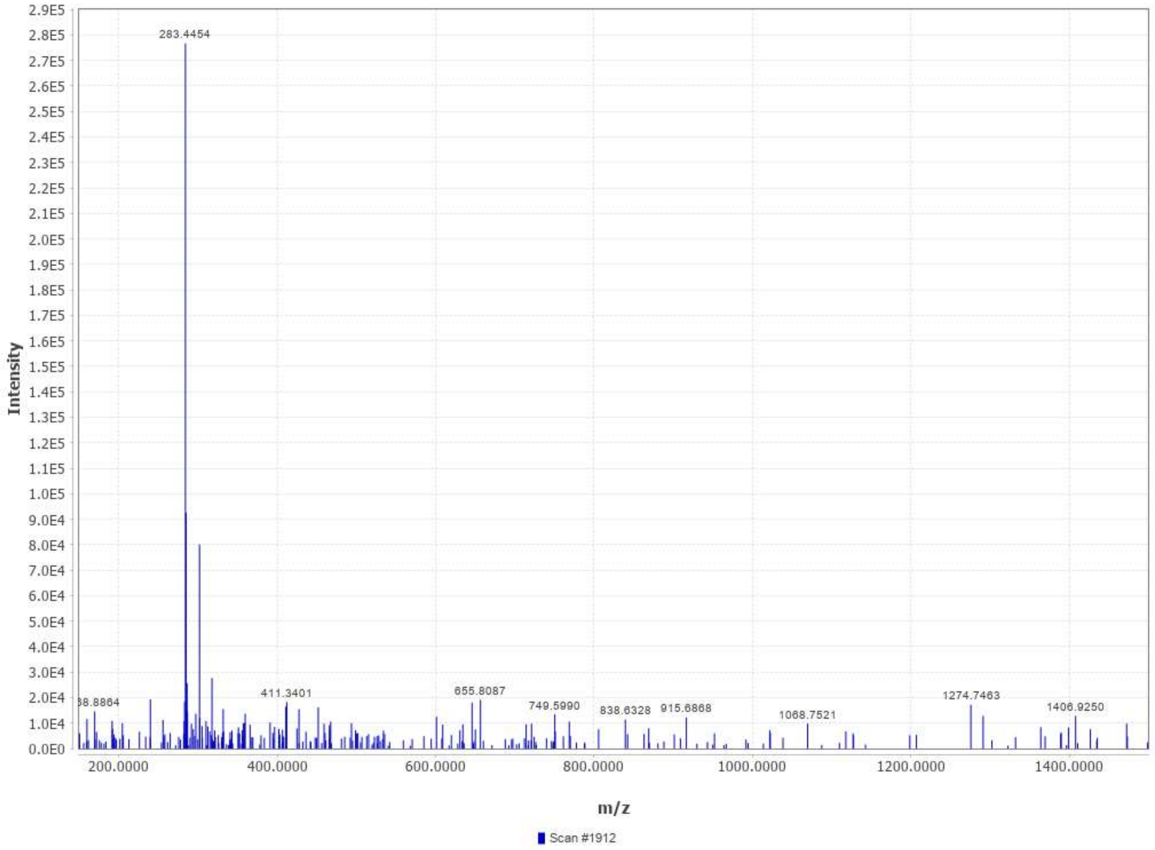

Xanthosine-5′-monophosphate disodium salt

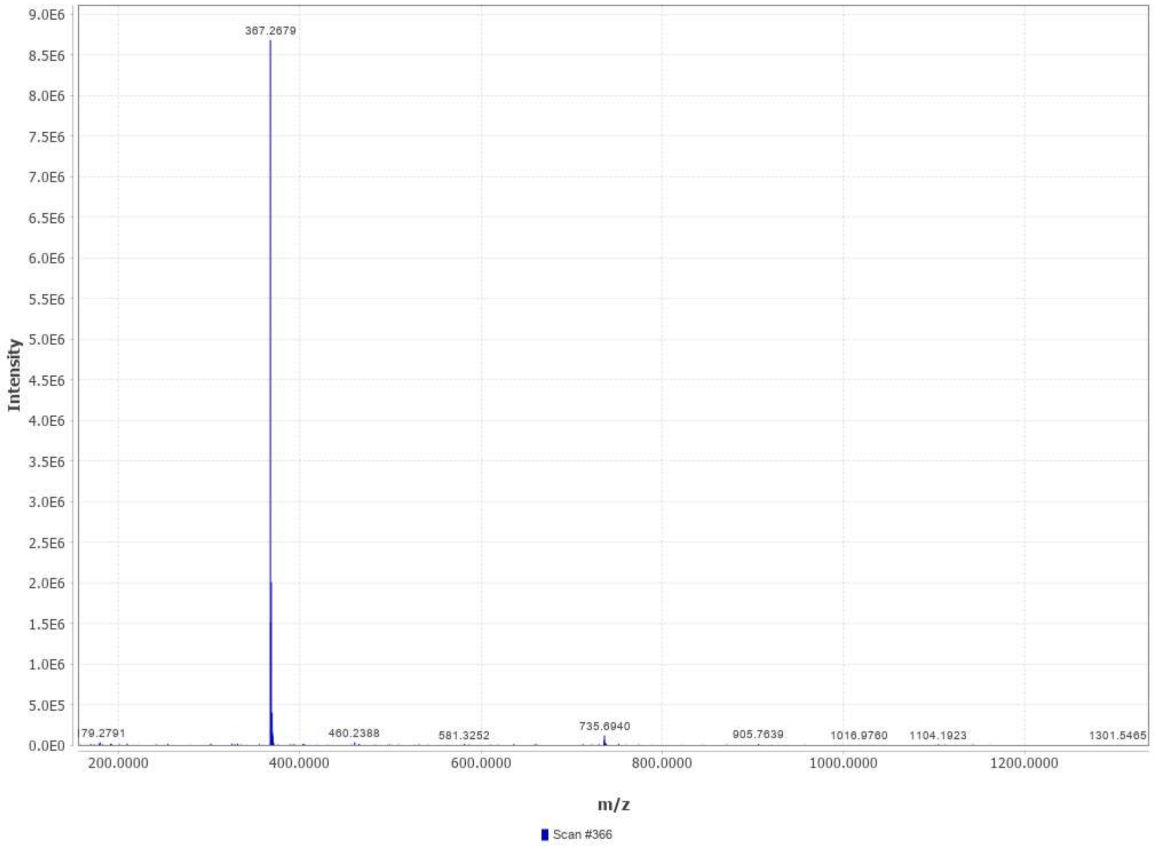

Supplement: Supplementary file 1 [file life-13-01435-s001.zip › life-2440815-supplementary.pdf]
